# Possible molecular mechanisms underlying the development of atherosclerosis in cancer survivors

**DOI:** 10.3389/fcvm.2023.1186679

**Published:** 2023-06-02

**Authors:** Priyanka Banerjee, Julia Enterría Rosales, Khanh Chau, Minh T. H. Nguyen, Sivareddy Kotla, Steven H. Lin, Anita Deswal, Robert Dantzer, Elizabeth A. Olmsted-Davis, Hung Nguyen, Guangyu Wang, John P. Cooke, Jun-ichi Abe, Nhat-Tu Le

**Affiliations:** ^1^ Center for Cardiovascular Regeneration, Department of Cardiovascular Sciences, Houston Methodist Research Institute, Houston, TX, United States; ^2^ Department of Cardiology, The University of Texas MD Anderson Cancer Center, Houston, TX, United States; ^3^ School of Medicine, Instituto Tecnológico de Monterrey, Guadalajara, Mexico; ^4^ Department of Life Science, University of Science and Technology of Hanoi, Vietnam Academy of Science and Technology, Hanoi, Vietnam; ^5^ Department of Symptom Research, The University of Texas MD Anderson Cancer Center, Houston, TX, United States; ^6^ Cancer Division, Burnett School of Biomedical Science, College of Medicine, University of Central Florida, Orlando, FL, United States

**Keywords:** senescence, atherosclerosis, disturbed flow, sumoylation, endoMT

## Abstract

Cancer survivors undergone treatment face an increased risk of developing atherosclerotic cardiovascular disease (CVD), yet the underlying mechanisms remain elusive. Recent studies have revealed that chemotherapy can drive senescent cancer cells to acquire a proliferative phenotype known as senescence-associated stemness (SAS). These SAS cells exhibit enhanced growth and resistance to cancer treatment, thereby contributing to disease progression. Endothelial cell (EC) senescence has been implicated in atherosclerosis and cancer, including among cancer survivors. Treatment modalities for cancer can induce EC senescence, leading to the development of SAS phenotype and subsequent atherosclerosis in cancer survivors. Consequently, targeting senescent ECs displaying the SAS phenotype hold promise as a therapeutic approach for managing atherosclerotic CVD in this population. This review aims to provide a mechanistic understanding of SAS induction in ECs and its contribution to atherosclerosis among cancer survivors. We delve into the mechanisms underlying EC senescence in response to disturbed flow and ionizing radiation, which play pivotal role in atherosclerosis and cancer. Key pathways, including p90RSK/TERF2IP, TGFβR1/SMAD, and BH4 signaling are explored as potential targets for cancer treatment. By comprehending the similarities and distinctions between different types of senescence and the associated pathways, we can pave the way for targeted interventions aim at enhancing the cardiovascular health of this vulnerable population. The insights gained from this review may facilitate the development of novel therapeutic strategies for managing atherosclerotic CVD in cancer survivors.

## 1. Introduction

As individual age, the prevalence of various disorders and diseases, including atherosclerotic CVD, increases in the population (1). Atherosclerotic CVD remains the leading cause of morbidity and mortality worldwide, particularly in those aged 65 and older (2, 3) (see Table 1). Various scoring systems, such as the Pooled cohort equations (PCE), Framingham risk score, and Reynolds Risk Score, have been utilized to assess the risk of atherosclerotic CVD. These scoring systems consistently indicate that age is a significant risk factor for atherosclerotic CVD, with both females and males having a greater risk of CVD as they age (see Table 2).

**Table 1:** The heart disease statistics in different age groups in United States (national health and nutrition examination survey) (4).

Age group (years)	Coronary heart diseases (CHD) (% of population)		Myocardial infarction (MI) (% of population)	
	Male	Female	Male	Female
20–39	0.6	0.9	0.4	0.4
40–59	6.9	6.6	3.2	1.9
60–79	22.0	13.4	12.6	4.5
80+	33.9	21.6	15.8	8.7

**Table 2:** Framingham risk score for risk of CVD with age and sex (5).

Age group (years)	Risk points, male	Risk point, female
<34	−1	−9
35–39	0	−4
40–44	1	0
45–49	2	3
50–54	3	6
55–59	4	7
60–64	5	8
65–69	6	8
70–74	7	8

Cancer survivors who have undergone cancer treatment, including radiation therapy, face an elevated risk of developing atherosclerotic CVD, with a 1.3–3.6 fold increase in developing coronary artery disease (CAD) and a 1.7–18.5-fold increase in developing atherosclerotic risk factors, which may lead to potentially fatal consequences (6, 7). A cross-sectional study conducted by the National Health and Nutrition Examination Survey (NHANES) assessed the 10-year risk of atherosclerotic CVD using the Pooled Cohort Equations in both cancer survivors and non-cancer patients. The study found that the risk of atherosclerotic CVD was higher in cancer survivors compared to non-cancer patients (8) (see Table 3). This increased prevalence and mortality has been linked to cancer therapies, including IR and chemotherapeutic agents (2).

**Table 3:** The 10-year risk of atherosclerotic CVD in cancer survivors (8).

Cancer types	Odds ratio (95% confidence intervals)
Testicular cancer followed by	11.47 (1.13–116.51)
Prostate cancer	9.45 (4.53–19.73))
Bladder/Kidney cancer	7.27 (2.58–20.40)
Melanoma	5.84 (2.68–12.73)
Lung	5.03 (1.71–14.80)
Colorectal	3.72 (1.03–13.46)
Breast	1.95 (0.99−3.86)
Cervical	0.81 (0.29–2.24)

Cellular senescence, a hallmark of aging, occurs when cells cease to divide in response to various stress stimuli, both internal and external. These stimuli include d-flow, oncogene activation, DNA damage, mitochondrial dysfunction, reactive oxygen species (ROS), and cancer treatments including radiation therapy and chemotherapy. Recent study by Milanovic and colleagues has shown that chemotherapy may induce senescence in cancer cells, which can have negative effects on tissue functions due to the secretion of various factors that regulate vital biological processes, such as cellular metabolism, cell growth, and inflammatory signaling (9, 10). This secretion of factors known as senescence-associated secretory phenotype (SASP). These senescent cancer cells can also escape cell cycle arrest and apoptosis, leading to an increase in their clonogenic growth potential. This phenomenon is known as SAS (11–16).

Milanovic and colleagues' seminal observation revealed clear differences between replicative senescence (RS) and stress-induced premature senescence (SIPS) (11–16). SIPS is not necessarily associated with telomere shortening, unlike RS. Additionally, SIPS may promote cancer treatment resistance, tumorigenesis, and numerous age-related disorders, including atherosclerotic CVD in cancer survivors (13–20).

Cancer treatment can also lead to changes in the structure and function of the vasculature, including alterations in the morphology and function of ECs that can induce EC senescence (7). EC senescence can result in changes in the hemodynamics, structure, and function of the vasculature (21–23) and these alterations are driven by a complex interplay of molecular and cellular mechanisms. These mechanisms include DNA damage response (DDR), Shelterin disruption, aberrant post-translational modifications, signaling pathways, elevated generation of ROS, and sustained activation of pro-inflammatory and pro-fibrotic transcription factors (21, 24, 25), all of which can cause a positive feedback loop contributing to the sustained inflammation, which can explain the elevated risk of atherosclerotic CVD in cancer survivors.

In this review, we aim to provide a comprehensive understanding of the possible molecular mechanisms regulating the development of atherosclerotic CVD in cancer survivors to establish rational therapeutic directions based on a mechanistic understanding of their interactions and interplay between cancer therapy and hemodynamics. We will summarize the similarities and differences between RS and SIPS, the latter of which plays a critical role in the induction of the SAS phenotype. We will describe the importance of the regulatory mechanisms for telomere DNA protection, as telomere DNA damage and dysfunction are critical for the induction of SIPS. We will also examine the key pathways involved in cellular senescence. Finally, we will discuss potential therapeutic interventions that may benefit patients with atherosclerotic CVD, with a focus on the BH4 pathway.

Our goal is to provide current knowledge to help future studies understand how cellular senescence is triggered in ECs in response to cancer treatments and how a distinct type of SASP (SAS) in ECs may contribute to the development of atherosclerotic CVD in cancer survivors.

Additionally, we aim to highlight the potential of senolytic drugs in preventing, delaying, and alleviating various age-related disorders, including atherosclerotic CVD, in humans (26).

## 2. Cellular senescence has been implicated in the development of atherosclerosis and cancer

### 2.1. Atherosclerosis

Atherosclerosis is a chronic and progressive inflammatory disease that involves various risk factors, including d-flow and elevated level of oxidized low-density lipoprotein (oxLDL) (27). It can present in different clinical forms, such as CAD, ischemic heart disease, ischemic stroke, and peripheral arterial disease (PAD) (28). This disease typically begins in early life and has a long subclinical phase (2, 3). The aging process is a significant risk factor for atherosclerosis due to the deterioration of the balance between vasodilator and vasoconstriction factors secreted by ECs, which leads to vascular senescence and dysfunction. Cellular senescence have been shown to play a significant role in the development and progression of atherosclerosis (29). The accumulation of senescent cells during the aging process upregulates the expression of numerous molecules that fuel age-associated disorders, including atherosclerotic CVD (30–32).

Recent studies have also suggested that cellular senescence may play a role in plaque destabilization and rupture, leading to acute cardiovascular events. For instance, senescent vascular smooth muscle cells (VSMCs) within the plaque have been shown to have increased matrix metalloproteinase (MMP) activity, which can contribute to plaque rupture and thrombosis. Additionally, senescent cells can secrete SASP, characterized by the release of numerous molecules including proinflammatory cytokines and chemokines, such as interleukine 1β (IL1β), tumor necrosis factor α (TNFα), interferon γ (IFNγ), and transforming growth factor β (TGFβ). The SASP can lead to chronic inflammation by inducing the persistent pro-inflammatory senescence phenotype (PISP), promoting the recruitment and activation of immune cells within the plaque, impairing cholesterol efflux from macrophages, and leading to the accumulation of lipid-laden foam cells in the plaque (30–32).

### 2.2. Cancer

Cellular senescence can have a dual role in cancer, both suppressing and promoting its development and progression (33). On one hand, it can suppress cancer by halting the cell cycle of cells with DNA damage or mutations that could lead to cancer. On the other hand, senescent cells can also contribute to cancer development and progression by releasing SASP. The SASP can be beneficial by preventing cell division and promoting immune clearance of damaged cells, thus reducing the risk of tumor formation. However, the SASP can also promote inflammation, tissue remodeling, and angiogenesis, creating a microenvironment that is favorable for cancer cell growth and spread (34, 35).

Over time, the accumulate of senescent cells in tissues can lead to chronic inflammation, tissue dysfunction, and organ failure, all of which can increase the risk of cancer development (36). Several studies have shown that the presence of senescent cells in tissues is associated with a higher risk of cancer and poorer prognosis in cancer patients. Therefore, strategies aimed at eliminating senescent cells or modulating their SASP may have therapeutic potential for the prevention and treatment of cancer.

### 2.3. Cancer survivors

Milanovic and colleagues have demonstrated that chemotherapy can drive a specific subset of senescent cancer cells to reprogram and acquire a proliferative phenotype known as SAS. This unique state allows these cells to bypass cell cycle arrest and significantly enhances their growth and proliferation (9–16), independent of senescence-induced cell cycle arrest (17, 18) or cell death (13, 14). This adaptive mechanism may underlie the resistance of cancer cells to chemotherapy and radiation therapy, enabling their survival and proliferation even in the presence of treatment (13, 14, 19).

These findings highlight the significant role of cellular senescence in the development and progression of atherosclerotic CVD and cancer, including among cancer survivors. However, it is important to note that senescence is also associated with impaired angiogenesis (37). Studies have demonstrated that inhibiting telomerase in ECs can reduce angiogenesis in tumor (38) and animal model of hind limb ischemia (39). Therefore, targeting senescent cells could be a promising therapeutic strategy for managing these diseases. Additionally, while cancer treatment can induce EC senescence (40), it remains unclear whether the SAS phenotype in ECs expressing SASP contributes to the delayed onset of atherosclerotic CVD following cancer treatment. Further studies are needed to investigate this aspect and shed light on the interplay between EC senescence, SAS status, and the development of atherosclerosis in cancer survivors.

## 3. Cellular senescence

### 3.1. Cellular senescence is related to telomere shortening

Cellular senescence is a state of irreversible cell cycle arrest, first described by Hayflick and Moorhead in 1961 when they observed that human diploid fibroblasts underwent this process after serial passaging. Successive cell division can shorten telomeres to a critical length, known as the Hayflick limit (41–43), at which point they are unable to form a t-loop, causing cells to become senescent (44–47). This type of growth arrest was later named RS (48–50).

Telomeres are repetitive hexanucleotide 5′-TTAGGG-3′tandem sequences that cap the chromosome ends, responsible for maintaining genomic stability (41–43). On eukaryotic chromosomes, they span approximately 3–10 kb and end with a 3′-end single-stranded overhang (51). After each round of replication, the ends of chromosomes resemble damaged DNA, triggering DDR. This leads to the inability to fully replicate DNA strands, causing telomeres to shorten by about 50–200 base pairs after each cell division (44–47).

To prevent continuous shortening of telomeres, the telomerase protein, which is a telomere-specific ribonucleoprotein, adds single-stranded telomeric repeats to the chromosomal 3′ ends (52). However, many human somatic cells lack telomerase, including human dermal fibroblast cells. Therefore, during cell division, the telomere length progressively shortens. After a certain time, the telomeres reach a critical length, and a DDR signal triggers senescence in the cells. This can cause cell cycle arrest and cellular senescence by upregulating p53-mediated p21 transcription (53–55).

Cellular senescence is related to shortening of telomeres to the Hayflick limit and detected through upregulated expression of senescence associated β-galactosidase (SaβG) and cyclin-dependent kinase inhibitors p21^Cip1^ (or p21^Waf1^) and p16^INK4a^ (56, 57). This process can lead to loss of tissue homeostasis and increased susceptibility to age-related disorders and diseases, including atherosclerotic CVD and cancer. Poorer survival rates have been reported in individuals over 60 years of age with shorter telomeres compared to their younger counterparts (58).

### 3.2. Cellular senescence can occur independently of telomere shortening

Telomere shortening has been linked to cellular senescence (59), but the relationship between these two is complex (60–62). Telomere shortening that occurs after successive cell divisions can lead to RS, which prevents further replication (44–47). However, cellular senescence can also occur independently of telomere shortening (52), indicating that telomere shortening alone may not be the sole cause of cellular senescence. This offers a significant opportunity for further studies. For instance, ventricular stiffness and impaired cardiac function can occur during aging due to cardiomyocyte hypertrophy and fibrosis, although the relationship between cardiac hypertrophy, fibrosis, and telomere length remains unclear (63). Cells with telomerase expression, such as human keratinocytes, epithelial cells, and rodent cells, can maintain long telomeres in culture but still undergo senescence (52, 64). Telomere attrition, but not the inherently short leukocyte telomere length at birth, has been found to be the main cause of leukocyte telomere shortening and correlates with telomere length in atherosclerotic CVD patients (65).

Additionally, studies have shown that cellular senescence is also a characteristic of chronic stress, which can provoke premature senescence, or so-called SIPS through internal and external stimuli (66–69). SIPS can occur independently of telomere shortening, although telomere shortening may be related to SIPS. For instance, SIPS can be induced in immortalized human foreskin fibroblasts expressing telomerase (hTERT-BJ1) exposed to hydrogen peroxide or Ultraviolet B (45). Another example is the induction of SIPS in renal tubular cells exposed to the urine of patients with calcium oxalate kidney stones, likely due to oxidative stress induced by oxalate and calcium oxalate monohydrate (70).

**Figure 1:**
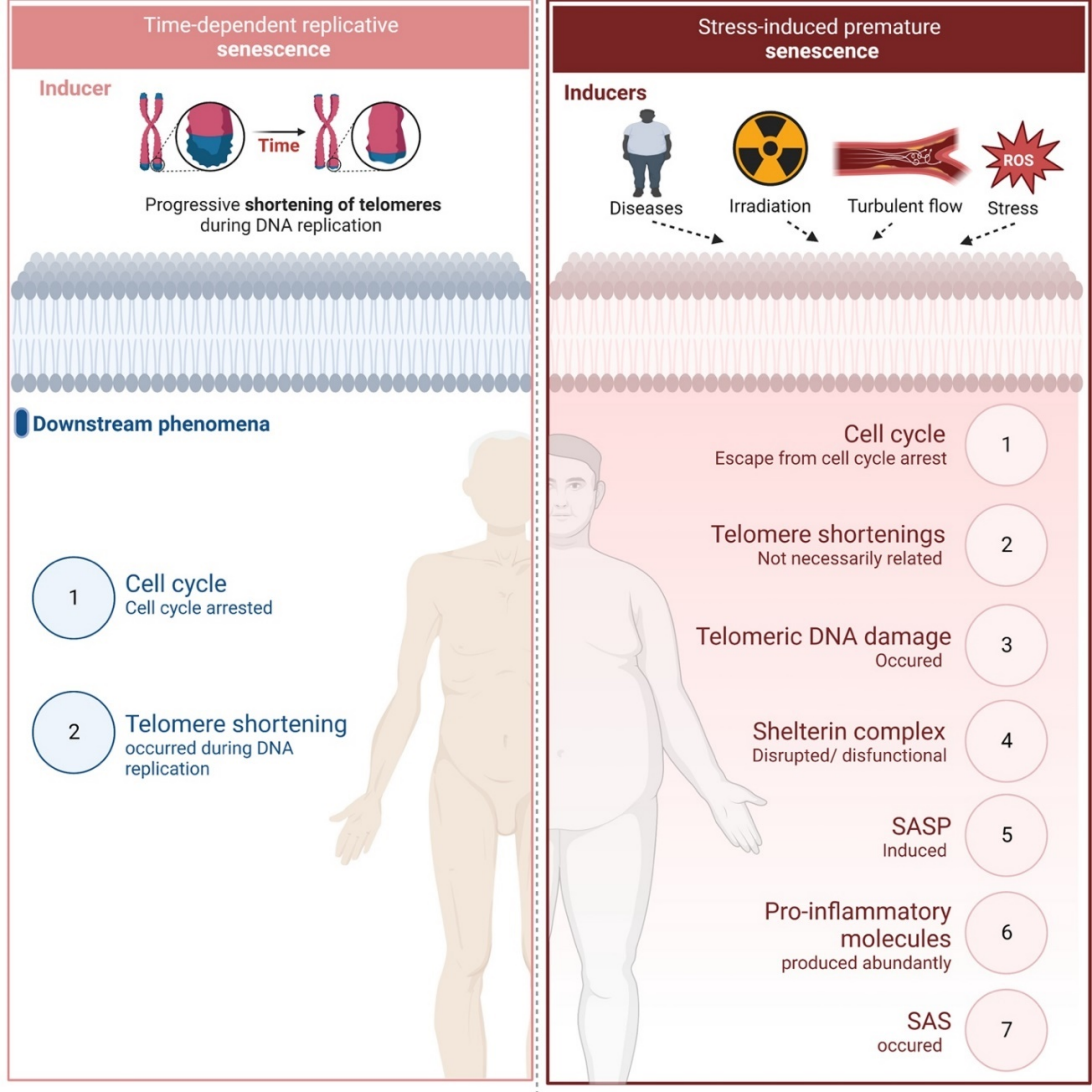
Replicative senescence vs stress-induced premature senescence. Different inducers and down-stream effects of two types of senescence (All the figures were made in Biorender.com).

RS and SIPS are induced at different time frames and regulated by different mechanisms as reviewed elsewhere (44–47, 71). Figure 1 provides a summary of the mechanisms involved.

### 3.3. Telomeric DNA damage can induce PISP

DNA damage, as detected by the formation of g-H2AX foci (g-foci), has been linked to cellular senescence and aging at the organismal level. Both telomeric and non-telomeric DNA damage have been implicated in cellular senescence. Nakamura and colleagues have shown that g-foci related to senescence can be found at uncapped telomeres or non-telomeric DNA damage sites on chromosomes in both humans and mice (13). Telomeric DNA is vulnerable to oxidation due to the low redox potential of guanine (72). However, repair of telomeric DNA damage is less effective and much slower than repair of genomic DNA damage, which occurs within 24 h (73–75). This leads to long-lasting telomeric DNA damage signaling that can induce the delay and persistent DDR at telomeres for months (76), resulting in the formation of telomere-associated DDR foci (TAF) (63). This process occurs in aging post-mitotic cells, such as cardiomyocytes and neurons, even though these cells have relatively longer telomeres (73). These observations suggest that stress-induced telomeric DNA damage and the persistent DDR and subsequent TAF formation, not telomere shortening, can induce PISP (77–79).

### 3.4. The SASP has been implicated in both atherosclerosis and cancer

Senescent cells, including both RS and SIPS, are known to secrete a variety of molecules that contribute to pro-inflammatory and pro-tumorigenic environment, collectively referred to as SASP (10, 13, 15, 80–84). These molecules include pro-inflammatory cytokines, chemokines, growth factors, pro-angiogenic factors, small molecules, lipids, ROS, and proteases (69, 80, 85). The SASP is reversible, as suggested by Coppé and colleagues (17). Unlike apoptotic and quiescent cells, SASP cells remain metabolically active and have been found to express significantly higher levels of glycolytic pathway enzymes such as hexokinase, phosphoglycerate kinase, and phosphoglycerate mutase, as well as higher glycolytic activity compared to young cells (77, 86, 87). Furthermore, mitochondrial ROS (mtROS) production and succinate induction are upregulated in SASP cells, even when both oxidative phosphorylation (OXPHOS) and glycolysis are inhibited by low dose IR without necrosis or apoptosis (88).

In addition to their effects of neighboring cells, SASP cells can also communicate via extracellular vesicles (EVs) (89). Activated senescent cells can generate more functional Evs than non-senescent cells by upregulating p53 expression, and these Evs can upregulate ROS induction and promote senescence in neighboring cells (90, 91).

### 3.5. SAS, a distinct type of SASP, has been found to contribute to the lasting effects of chemotherapy

Milanovic and colleagues reported that cancer therapy-induced senescence (TIS) can trigger the SAS proliferative phenotype in cancer cells, allowing them to evade senescence-induced cell cycle arrest and exhibit enhanced clonogenic growth potential (15, 16, 20). SAS is regulated independently of cell cycle arrest (17, 18) and cell death (13, 14, 92) and is considered a critical mechanism in the development of resistance to cancer therapy (13, 14, 19, 92, 93).

In addition to TIS, oncogene-induced senescence (OIS) is another phenomenon in which oncogenes are activated in non-tumor cells, resulting in a stable cell cycle arrest (85). Leon and colleagues have found that OIS and overexpression of the oncogene HRASG12V in IMR90 cells increase active histone H3K79 di- and tri-methylation (H3K79me2/3) at the ILIA gene locus (94). The histone methyltransferase disruptor of telomeric silencing 1-like (DOT1l) regulates the increase in H3K79me2/3 occupancy at the IL1A gene locus, which is critical for IL1A expression and gene expression regulation during OIS. However, while DOT1l is an epigenetic regulator of the SASP, its depletion does not affect OIS-induced cell cycle arrest, suggesting that it is not involved in SAS. Although epigenetic changes may lead to irreversible SASP in OIS cells (95, 96), their role in SAS remains unknown, and additional stressors that can result in both SASP and SAS necessitate further study.

It is important to note that, although the findings observed by Milanovic and colleagues were made on cancer cells, it is possible that a similar phenomenon might also occur in vascular cells, including ECs. Both senescence and macrophage proliferation contribute to CVD (97), and therefore, the SAS phenotype in ECs may also play a role in CVD. Therefore, further investigation on the role of SAS in ECs is necessary to fully understand its contribution to atherosclerotic CVD.

### 3.6. DDR is a link between atherosclerosis and resistance to cancer therapy

As we discussed above, cellular senescence has been implicated in the development of both atherosclerosis and cancer including in cancer survivors. At the molecular level, cellular senescence is associated with both telomeric and non-telomeric DNA damage in cells of the vessel wall, including ECs, triggering the DDR pathway (98–100). DDR maintains genetic stability and cell integrity when exposed to DNA damaging agents, such as IR and chemotherapeutic drugs used in cancer treatments. DDR induces cell cycle arrest for DNA repair and promote apoptosis and senescence to prevent propagation of damaged DNA. However, dysregulation of DDR can lead to resistance of cells to cancer treatments, highlighting its potential as a target to enhance sensitivity to cancer therapies (101, 102).

The major DDR pathways are regulated by the ataxia-telangiectasia mutated and ataxia-telangiectasia and Rad3 related (ATM/ATR) pathways, which phosphorylate proteins at DNA damage sites, including histone H2A (H2AX), to form phosphorylated gH2AX. Additionally, ATM/ATR activate CHK2 and CHK1, respectively (103), which prevent cells with damaged DNA from entering mitosis, especially when cells have a defective G1 checkpoint. However, a defective checkpoint is common in cancer cells due to p53 mutations. CHK1 is a potential therapeutic target, and CHK1 inhibitors are being developed and used as single agents or in combination with IR or genotoxic chemotherapies in preclinical and clinical studies (104). As such, these pathways can be targeted for further study.

## 4. Mechanisms involved in the regulation of telomere protection pathways

Cellular senescence can occur independently of telomere shortening, highlighting the importance of understanding the mechanisms that protect telomeres from DNA damage and dysfunction to mitigate senescence-associated disorders and diseases. In this section, we will explore the mechanisms involved in the regulation of telomere protection pathways.

Chromosome ends must be shielded from damage to prevent the acceleration of cellular senescence, which would lead to activation of DDR and DNA damage repair machinery. This protection is accomplished by specific factors associated with telomeres, including telomerase, non-coding telomeric repeat-containing RNAs (TERRA), and Shelterin (55, 105, 106).

### 4.1. Telomerase

Telomerase, which is comprised of a ribonucleoprotein with an RNA subunit *TERC* and a reverse transcriptase enzymatic subunit TERT, catalyzes the addition of repetitive hexanucleotide 5′-TTAGGG-3′sequences to chromosome ends. This process known as telomere elongation (107–113). In addition to this primary function, telomerase can also inhibit immune cell apoptosis, protect neurons from oxidative stress, and regulate inflammatory responses (114–119).

Furthermore, TERRA, a class of long noncoding RNAs transcribed at telomeres, is also suggested to participate in the protection of chromosome ends and telomeres. However, the mechanisms of telomere protection regulated by TERRA are yet to be fully elucidated (120). Additionally, the Shelterin can shape, safeguard, and protect telomeres during proliferation.

### 4.2. Shelterin

**Figure 2:**
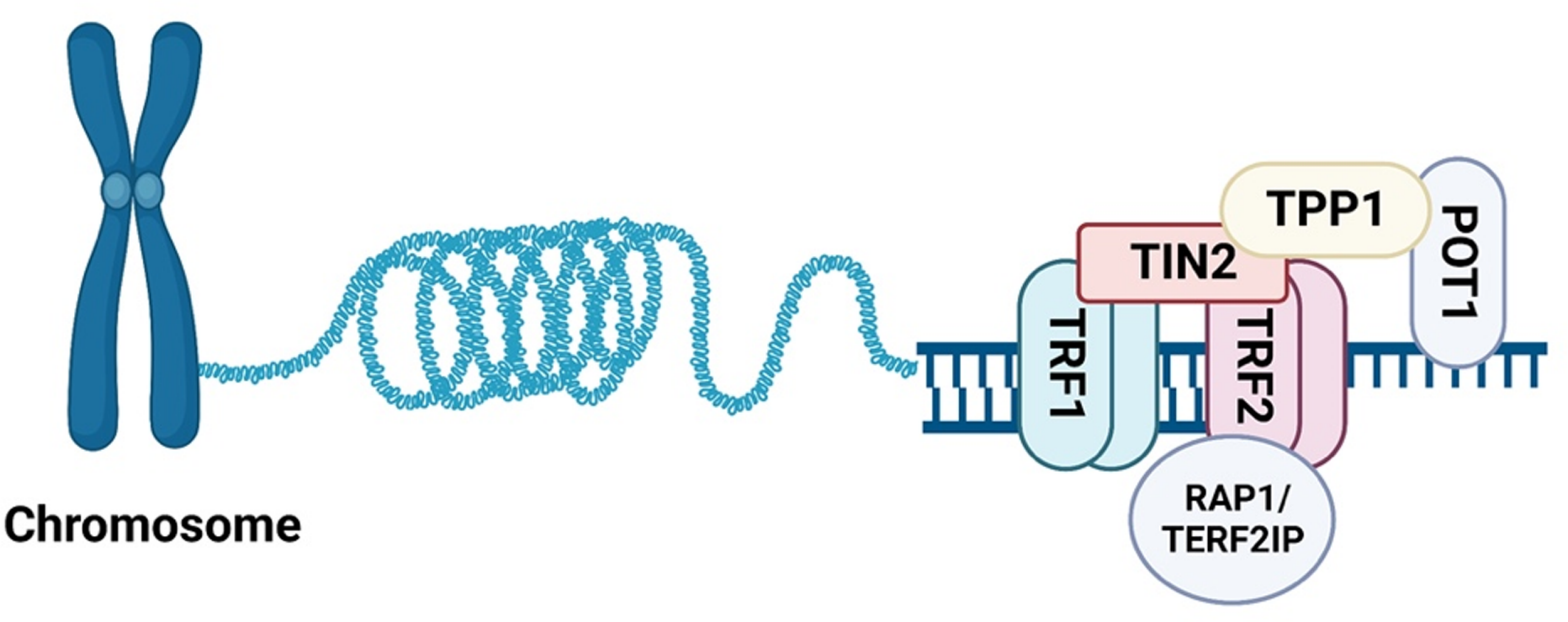
Assembling of Shelterin. The six known components of Shelterin: TRF1, TRF2, TERF2IP (RAP1), TIN2, TPP1, and POT1. TRF1 and TRF2 bind to telomeric DNA duplexes, while POT1 binds to single-stranded DNA in the 3′ overhang region. TERF2IP binds TRF2 and does not directly bind DNA (All the figures were made in Biorender.com).

In addition to telomerase and TERRA, the Shelterin complex can shape, safeguard, and protect telomeres during proliferation. The Shelterin complex is comprised of six proteins: TERF1, TERF2, POT1, TIN2, TPP1, and TERF2IP, as illustrated in Figure 2. Its primary function is to safeguard telomeres by preserving their structure and function, ensuring genomic stability (121, 122). In the following sections, we will examine the specific roles of each Shelterin protein, and explore recent studies on the emerging functions of post-translational modifications of TERF2IP in the context of telomere protection, cellular senescence, and potential implications in atherosclerotic CVD.

Telomere repeat-binding factor 1 and 2 (TRF1 and TRF2) are two key components of vertebrate telomeres that share the TRF homology domain (TRFH) and bind double-stranded telomeric DNA as homodimers. TRFH on TRF1 and TRF2 is critical for TRF1-TRF2 dimerization, DNA binding, telomere localization, and modulation of TRF1-TRF2 interaction with other Shelterin proteins (123). To study how TRF1 and TRF2 locate TTAGGG repeats on DNA tightropes and assemble the Shelterin complex, Lin and colleagues used single-molecule fluorescence imaging to observe the dynamics of quantum dot (QD)-labeled TRF1 and TRF2 on λDNA and DNA substrates containing alternating regions of telomeric and non-telomeric sequences. They observed that TRF1 directly binds telomeric sequences with little 1D searching on non-telomeric DNA, while TRF2 extensive 1D searches on non-telomeric DNA through 1D sliding to find protein partners for assembling the Shelterin complex and stabilizing their interaction with specific telomeric DNA (124).

Besides their central role in telomere capping, elevated TRF2 expression is frequently found in tumors. El Mai and colleagues have demonstrated that TRF2 is expressed in the vasculature of most human cancers, where it colocalizes with the Wilms' tumor suppressor (WT1). TRF2 acts as a transcriptional target of WT1 and plays an essential role in EC proliferation, migration, and tube formation. Mechanistically, TRF2 binds and transactivates the promoter of angiogenic tyrosine kinase platelet-derived growth factor receptor β (PDGFRβ) through a mechanism distinct from that of telomere capping (125).

TRF1-interacting protein 2 (TIN2) is a key protein in the Shelterin complex, acting as a central mediator for TRF1 function. Using TRF1 as bait for interaction cloning, Kim and colleagues demonstrated that TIN2 interacts with TRF1 and co-localizes with it in the nucleus during metaphase chromosomes. TIN2 also binds telomere protection protein 1 (TPP1)/protection telomeres 1 (POT1) to regulate telomere length, telomeric capping, and telomerase activity (122, 126). POT1 (or POT1a and POT1b in rodents) is a single-stranded-DNA binding protein that plays a crucial role in telomeric capping and protection (122). In addition to modulating TRF1-TRF2 dimerization, TIN2 also acts as an adaptor protein that links TPP1/POT1 to TRF1-TRF2 on double-stranded telomeric DNA (127).

TTP1/POT1 are single-stranded telomeric DNA binding proteins that play essential roles in preventing the activation of the ATM/ATR pathways at telomeres (128–131). Although POT1a can block the binding of replication protein A (RPA) to telomeres, the binding affinities and abundance of TPP1/POT1a and RPA suggests that TPP1/POT1a is unlikely to exclude RPA, the major protein that binds single-stranded DNA. Takai and colleagues demonstrated that TIN2 deletion triggers the loss of telomere TPP1/POT1a, accumulation of RPA, and ATR activation, accompanied by all phenotypes provoked by POT1a/b deletion. While TIN2 has a minor role in TRF2-induced inhibition of ATM pathways (but not TRF2-induced inhibition of telomere fusions), it has a key role in TTP1/POT1-dependent inhibition of ATR pathways via stabilizing TPP1/POT1a on single-stranded telomeric DNA. This stabilization allows for effective exclusion of RPA and thus prevents ATR activation (128, 130, 131). Together, these observations indicate that TIN2 inhibits ATM/ATR, the two major DNA damage repair pathways, by stabilizing TRF2 on double-stranded telomeric DNA and stabilizing TPP1/POT1 on single-stranded-DNA overhang, respectively (130–133).

Of the six Shelterin proteins, TRF1-TRF2 bind double-stranded telomeric DNA through the C-terminal Myb domain and recruit TERF2IP, TIN2, TPP1, and POT1 for assembling the Shelterin complex (41). Among the six Shelterin proteins, TERF2IP, also known as repressor activator protein 1 (RAP1), is one of the most conserved proteins and directly binds TRF2 (55, 106, 122, 134).

The Shelterin complex is a promising target for chemotherapeutics. Table 4 provides a summary of the chemotherapeutics that have been used to target the Shelterin complex (135) (see Table 4).

**Table 4:** Chemotherapeutics targeting Shelterin complex (135).

Name of the chemotherapy	Cancers	Targeted Shelterin complex on telomere
RHPS4 and derivatives	Brain tumor (136)	Delocalization of TRF2, POT1
BRACO19	Glioblastoma (137)	TRF1, TRF2, POT1, downregulation of POT1
Dihydroartemisinin (DHA)	Esophageal cancer (138)	TRF2 downregulation
Telomestatin	Fibrosarcoma (*in vitro* study) (139)	Inhibit POT1 binding to telemere
AKT inhibitors	Glioblastoma (140)	Inhibition of TRF1 phosphorylation

#### 4.2.1. TRF1 and TRF2

To bind double-stranded telomeric DNA, TRF1 and TRF2 associate with TIN2, which stabilizes the telomeric localization of TRF1 and TRF2. Although TRF1 and TRF2 share similarities in structure and form TRF1-TRF2 dimers, their key functions are distinct from each other (141, 142). TRF1 negatively regulates telomerase-mediated telomere elongation to maintain telomeric homeostasis (143, 144). TRF1 depletion revealed TRF1 is a regulator of Shelterin localization on telomeres and maintenance of telomere functional structures. Martínez and colleagues have shown that conditional deletion of TRF1 in mouse embryonic fibroblasts is sufficient to induce severe telomeric damage without telomere shortening. These cells, in the absence of TRF1, rapidly underwent cellular senescence and had increased telomeric γH2AX foci and activation of the cell-cycle checkpoint kinase 2 and 1 (CHK2 and CHK1), which are known downstream targets for the ATM/ATR pathways, which is known as the major DDR pathways (145). Chromosomes with longer telomeric ends allow more TRF1 proteins to bind to telomeres, thereby inhibiting telomerase activity and suppressing telomere elongation. After multiple rounds of DNA replication, telomeric ends become shorter, which decreases TRF1 binding to telomeric ends and allows telomerase to activate telomere elongation. In aged ECs, both TRF1 mRNA and protein expression are decreased compared to that of young ECs (146–148).

TRF2, on the other hand, functions as a telomere capping protein and is involved in protecting chromosome ends from being recognized as DNA damage site, which can lead to genomic instability. In addition to its role in telomere attachment to nuclear membrane (149), TRF2 also plays a critical role in preventing telomere fusion and recombination. TRF2 depletion evokes telomere uncapping, leading to the activation of telomere dysfunctional DDR and induction of cellular senescence and oxidative stress, independent of telomerase activity (150). Specifically, TRF2 depletion rapidly induces end-to-end fusion, cellular senescence (147, 148), and cell death by activating the ATM/ATR pathways (145, 151, 152). In patients with heart failure, low TRF2 expression levels are associated with telomere shortening in cardiomycytes (153). These observations all together have demonstrated critical roles for both TRF1 and TRF2 in senescence and genomic instability (122, 154).

#### 4.2.2. TIN2

TIN2 serves as an adaptor protein for TRF1 and helps in stabilizing the telomeric localization of TRF1 and TRF2 (126). Additionally, TIN2 interacts with TPP1 through its TPP1 interacting domain, which connects TIN2 to POT1, facilitating the telomeric localization of the TPP1-POT1 complex (122). TIN2 contains a mitochondrial localization domain at its N-terminus (155, 156), allowing it to translocate to mitochondria, where it undergoes post-translational modifications and regulates mitochondrial OXPHOS (155, 156). Depletion of TIN2 using short hairpin RNA (shRNA) prevents glycolysis and enhances OXPHOS, leading to a decrease in ROS synthesis (155). Conversely, TIN2 accumulation in mitochondria leads to an increase in ROS synthesis, promoting cellular senescence. Lee and colleagues have demonstrated that the RNA binding protein Human antigen R (HuR) binds to the 3′-untranslated region of TIN2 mRNA and inhibits TIN2 protein synthesis (157). During RS, diminished expression of HuR leads to increased stabilization of TIN2 mRNA, resulting in enhanced TIN2 protein synthesis, mitochondrial translocation, and acceleration of ROS synthesis and cellular senescence (157). Given the critical roles of mitochondrial metabolism and respiration in age- and stress-induced senescence (158, 159), the involvement of TNI2 in this process is of great interest and deserves further investigation.

#### 4.2.3. TPP1

TTP1 binds TIN2, which tethers TIN2 to POT1 (122) and enhances POT1's binding affinity to single-stranded telomeric DNA (160–162). POT1 then recruits telomerase to telomeric ends by interacting with the telomerase N-terminal OB domain and TERT, the telomerase catalytic subunit (162). Sirtuin 1, a nicotinamide dinucleotide (NAD^+^)-dependent deacetylase, have been shown by Chen and colleagues to protect mesenchymal stem cells from senescence by mediating TPP1 expression, suggesting TPP1's involvement in cellular senescence (163). Additionally, Min and colleagues suggested that suppression of TPP1 expression leads to mitochondrial dysfunction and deregulated mitochondrial-ribosome function, which leads to telomere deprotection and RS in human diploid fibroblasts (164).

#### 4.2.4. POT1

POT1 is considered a terminal transducer of TRF1-mediated telomere length regulation because it directly binds the 3′ single stranded G-overhang of telomeres, preventing telomerase access to telomeres (see Figure 2) (165). In addition to its telomere-protective functions, POT1 also binds to TPP1 and double-stranded DNA damage sites to suppress non-homologous end joining (NHEJ) and protect DNA from double-stranded breaks (166). Human POT1 has been shown to protect telomeric ends and regulate telomerase activity by displacing/replacing the G-quadruplex structure (167). POT1 mutations have been detected in various cancers including chronic lymphocytic leukemia, glioma, melanoma, angiosarcoma, and colorectal cancer (168). The most common mutations are found in the OB-fold domains at the N-terminus, which disrupts POT1 binding to telomeric ends (168). A recent study by Kelich and colleagues showed that a POT1 heterozygous mutation p(L259S) in a patient with idiopathic pulmonary fibrosis can drive telomere loss, telomere DNA damage, and pre-mature senescence (169). In mice, there are two homologues of human POT1, POT1a and POT1b, and knock-out studies suggested that POT1 is involved in protecting telomere single-stranded DNA from end-to-end fusion.

#### 4.2.5. TERF2IP

TERF2IP, also known as RAP1, plays a crucial role in several biological processes, including telomere protection, homology-directed repair (HDR) regulation, inflammation, and metabolism (170, 171). TERF2IP binds telomeres through its association with TRF2, which enhances TRF2 binding to telomeres (172, 173). The TRF2-TERF2IP complex prevents the telomeric localization of Poly [ADP-ribose] polymerase 1 (PARP1) and structure-specific endonuclease subunit (SLX4) and thereby inhibits homologous recombination (HR)-triggered telomere attrition. Deletion of TERF2IP and TRF2 leads to the recruitment of PARP1 and SLX4 to telomeres, resulting in the recruitment of additional HR factors such as DNA repair protein RAD51, Exonuclease I (EXOI), and the MRN complex to telomeres (172). The role of TERF2IP in the development of premature senescence has been investigated in progeroid mice (77, 174). TERF2IP protects telomeres from NHEJ in both yeast and humans (175, 176), independently of its association with TRF2 (176). In senescent human cells, TERF2IP prevents fusion of critically short telomeres, thereby protecting telomeres (177) and regulates sub-telomeric transcription. In senescent cells with critically short telomeres, TERF2IP re-localizes to sub-telomeric regions and binds to the promoters of a group of genes called new Rap1 targets at senescence (NRTS), promoting loss of histones and activating a cascade of other genes in NRTS (178, 179).

### 4.3. Nitric oxide (NO) activates telomerase and delays EC senescence and the role of tetrahydrobiopterin (BH4)

#### 4.3.1. NO signaling

NO has a crucial role in various physiological and pathological processes in the human body, including protecting telomeres from shortening, damage, and dysfunction by regulating telomerase activity, thus protecting cells from senescence. Additionally, NO can prevent DNA damage and oxidative stress, which can also contribute to telomere shortening and dysfunction. Exogenous NO has been demonstrated to delay EC senescence in culture, suggesting that telomere shortening can be modulated beyond the number of cellular divisions. The mechanism by which NO stimulates telomerase activity is not yet fully understood, but it may react with tissue-derived oxygen radicals, thereby reducing oxidative stress, which has been shown to accelerate EC senescence. Alternatively, NO may upregulate telomerase activity via transcriptional and/or posttranscriptional mechanisms (180).

NO has important cardioprotective and anti-inflammatory effects, as it inhibits vascular wall apoptosis and lipid oxidation, prevents VSMC growth, and inhibits white blood cell adhesion and platelet aggregation. Additionally, NO induces blood vessel dilation, which is a vital mediator of vascular tone (181, 182). The integrity of EC function, including their capability to proliferate and migrate, is essential for angiogenesis. Therefore, EC senescence and the subsequent reduction in their proliferative ability may contribute to compromised angiogenesis associated with age. Additionally, senescent ECs express adhesion molecules that promote neutrophil adhesion and inflammation, enhancing the chronic inflammatory process that contributes to the progression of atherosclerosis.

Conversely, endothelial NO is essential for angiogenesis and protects against atherosclerosis. However, the bioavailability of endothelial-derived NO is impaired with aging, which may accelerate EC senescence, impairing EC function and contributing to impaired angiogenesis and atherosclerotic progression (180, 183–186).

#### 4.3.2. The impact of BH4 levels in anti-cancer treatment and their association with atherosclerotic CVD and the potential clinical applications

BH4 is an essential cofactor involved in several important enzymatic processes. It plays a vital role in the synthesis of neurotransmitters such as adrenaline, noradrenaline, serotonin, and dopamine, and participate in the degradation of phenylalanine (187). Additionally, BH4 serves as a cofactor for NOS3, which is responsible for the production of NO through the oxidation of L-Arginine to L-citrulline (188, 189). Supplementation with BH4 has been shown to have beneficial effects in improving conditions like hypertension and cardiac dysfunctions. By supporting NOS3 function, BH4 promotes the production of NO, thereby improving cardiovascular health (190).

However, under UV radiation or infrared exposure, BH4 can be oxidized to dihydrobiopterin (BH2), which can stimulate the production of higher levels of superoxide by eNOS. This oxidative process can lead to increased oxidative stress and potentially impact EC function (188). ECs have two pathways for synthesizing BH4: *de novo* synthesis from guanosine triphosphate (GTP) and a salvage pathway that involves recycling BH2 and quinoid-dihydrobiopterin (181, 191). These pathways are crucial for maintaining BH4 levels in ECs, which are essential for physiological processes and NO production, regulating EC function and vascular homeostasis.

**Figure 3:**
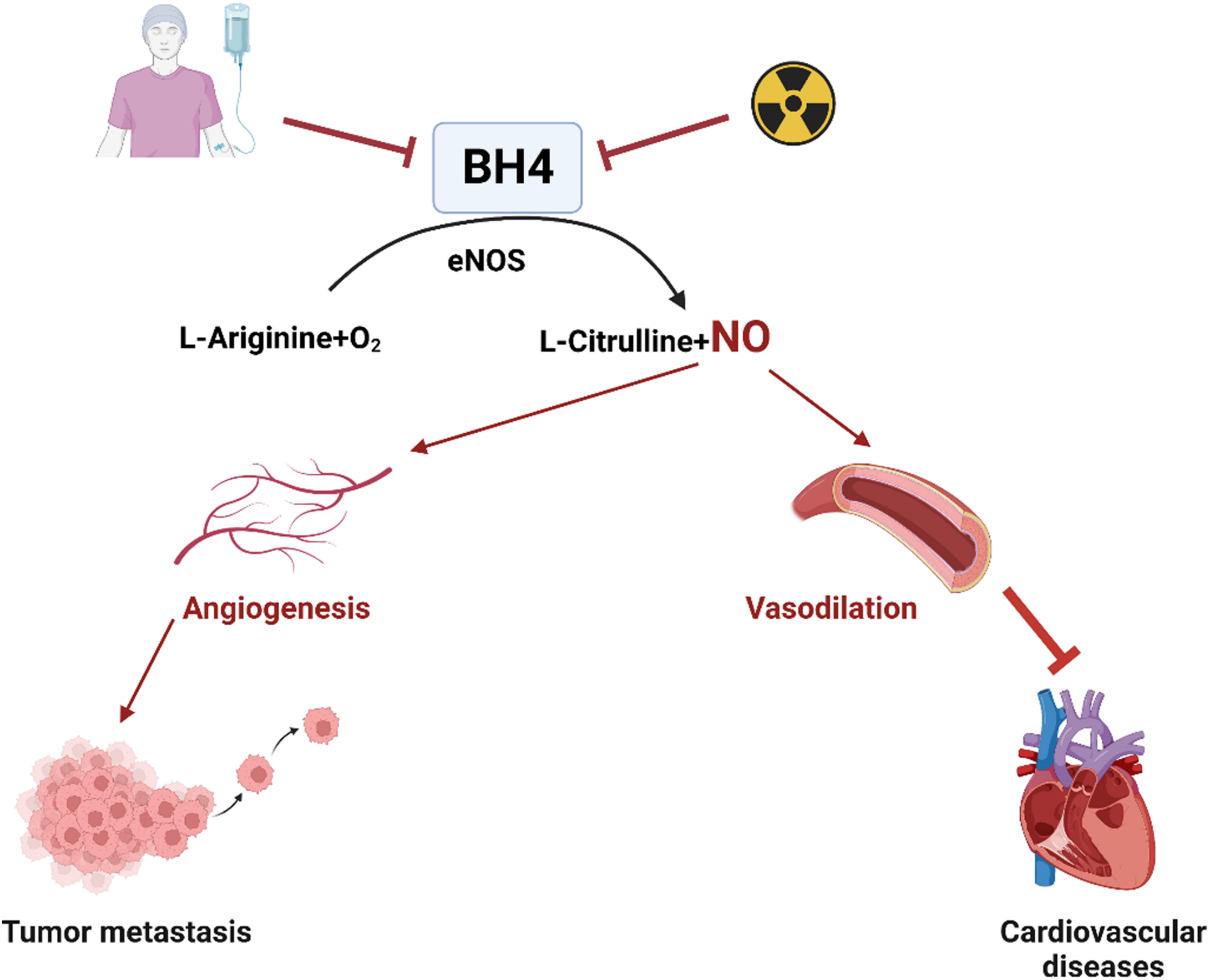
Chemotherapy or radiation therapy regulates BH4 levels and their association with atherosclerotic CVD (All the figures were made in Biorender.com).

Age-related studies have shown a decrease in vascular BH4 levels in animal models (192, 193), while in humans, aging has been observed to increase platelet and plasma BH2 levels without affecting BH4 levels or the BH4:BH2 ratio (182). This suggests increased oxidation of BH4 into BH2 with aging due to oxidative processes. Inhibiting the enzyme responsible for BH4 to BH2 conversion, such as with methotrexate treatment, leads to increased BH2 plasma levels, highlighting the importance of the BH4 recovery pathway for maintaining BH4 homeostasis (187, 194–196). Alterations in BH4 levels have been found to correlate with vascular dysfunction in ECs (188). In the context of tumor progression, BH4 promotes angiogenesis by activating eNOS for NO production (197). Conversely, inhibiting BH4 has been shown to attenuate tumor angiogenesis in a mouse model of hepatocellular carcinoma (198) (Figure 3). The changes in BH4 levels resulting from anti-cancer therapy and their potential impact on atherosclerotic CVD are summarized in Figure 3. These findings suggest that BH4 levels play a significant role in various physiological and pathological processes, including cancer progression and cardiovascular health. Further research is needed to fully understand the complex interactions and potential therapeutic implications of BH4 in cancer treatment and CVD.

## 5. The critical role of EC senescence in the development of atherosclerosis in cancer survivors

It is noteworthy that senescent ECs have been identified in human atherosclerotic plaques and have impaired function, such as reduced NO production and increased adhesion molecule expression, which can contribute to EC dysfunction and plaque formation (36, 174). The vascular system is crucial for maintaining health and survival (199, 200), and ECs plays a vital role in regulating vascular homeostasis. ECs line the luminal surface of blood vessels and dynamically respond to hemodynamic fluctuations to modulate blood flow. They also mediate the interaction of circulating blood components with the vessel wall and facilitate gas and nutrient exchange between the blood and tissues (71, 201). However, various stress stimuli, such as d-flow, increased production of ROS, DNA damage, mitochondrial dysfunction, exposure to inflammatory cytokines, cancer therapies, and activation of oncogenes, can induce EC senescence, adversely affecting these homeostatic functions, disrupting vascular integrity, and impairing their function (202).

The impairment of EC functions is an initial step in the progression of atherosclerotic CVD, which is linked to the aging process and a senescent phenotype in these cells. This can lead to disrupted permeability and pathological signaling cues, as evidenced by numerous studies (71, 203–205). Cancer therapy is a stress state that can induce EC senescence and subsequent vascular dysfunction, increasing the risk of atherosclerotic CVD (206). In ECs, therapy-induced senescence (TIS) is associated with chronic inflammation and the development of atherosclerotic lesions (11), which tend to occur in regions of the vasculature with d-flow (207). Notably, d-flow induces endothelial-to-mesenchymal transition (endoMT) through the transforming growth factor β (TGFß) signaling pathway (208), and this transition becomes pro-atherosclerotic through the deposition of fibronectin, increased expression of adhesion proteins, and recruitment of inflammatory cells (208).

Moreover, it is crucial to note that radiation therapy, a commonly cancer treatment, directly induces endoMT (209), which is accelerated by oxLDL and ultimately leads to atherosclerotic plaque formation (209). This endoMT not only leads to cardiovascular complications in cancer patient but also contributes to the development and progression of cancer-associated fibroblasts (CAFs) (210). It has been well-established that cancer and its therapies can induce or accelerate the aging process, which may explain the association between atherosclerotic CVD and cancer. Therefore, understanding the molecular mechanisms underlying EC senescence in atherosclerosis in cancer survivors is crucial to develop effective therapies for these conditions.

### 5.1. EndoMT

**Figure 4:**
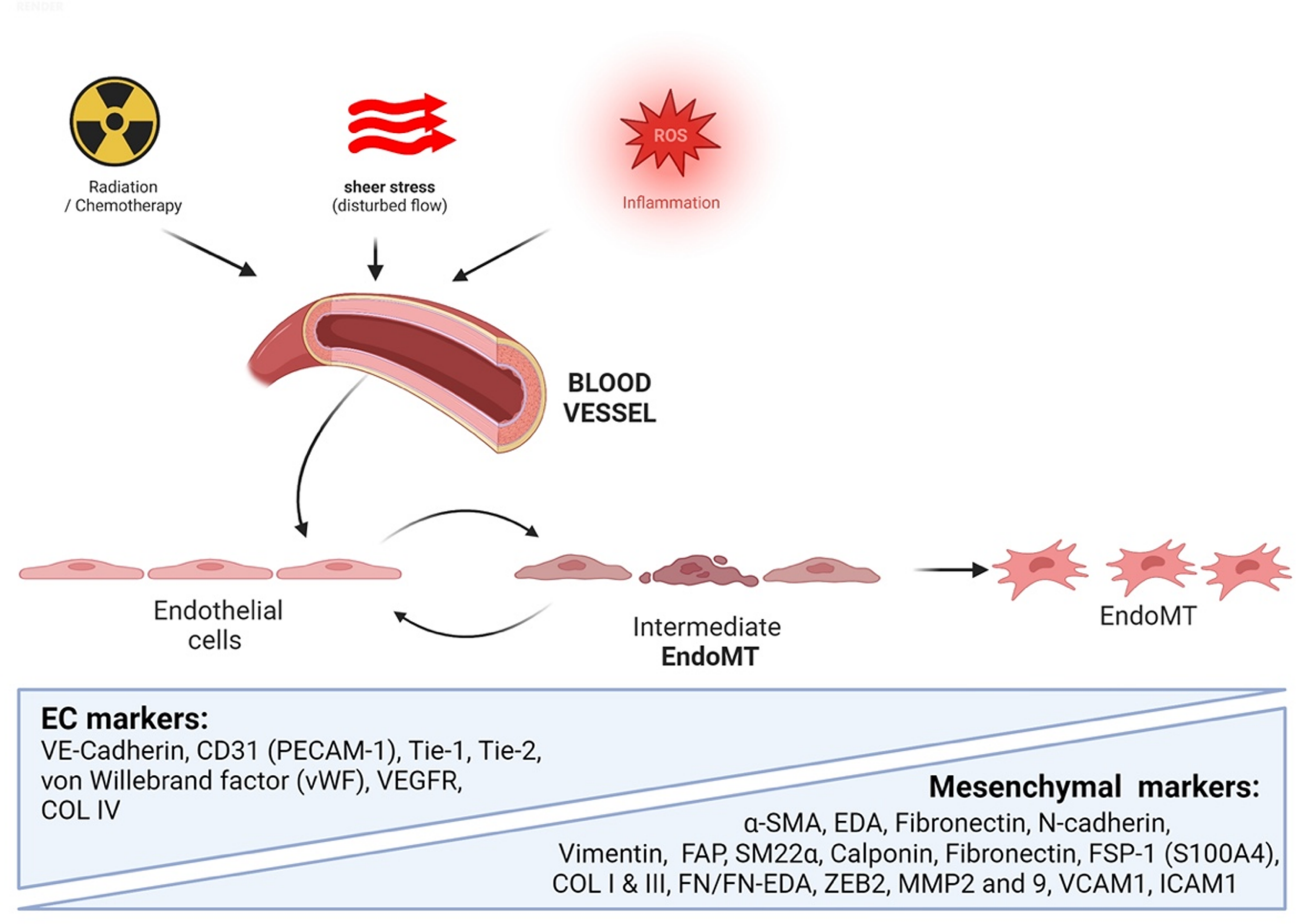
Extracellular stimuli induce endoMT. External stimuli such as radiotherapy, chemotherapy, d-flow, and inflammatory signals can trigger the transformation of ECs into mesenchymal cells, characterized by the enhanced expression of mesenchymal cell-specific markers (All the figures were made in Biorender.com).

ECs can undergo a process called endoMT, which leads to their trans-differentiation into mesenchymal cells (211, 212). During this process, ECs lose their characteristic features and acquire a mesenchymal phenotype. EC-specific genes such as Cluster of Differentiation 31 [CD31 or Platelet Endothelial Cell Adhesion Molecule 1 (PECAM1)], Vascular Endothelial Cadherin (VE-Cadherin or CDH5), Von Willebrand Factor (vWF), Tyrosine Kinase with Immunoglobulin-like and EGF-like domains 1 (TIE1), and TEK Receptor Tyrosine Kinase (TIE2) are downregulated (27, 211, 213), while mesenchymal cell-specific genes such as α-smooth muscle actin [αSMA or Smooth Muscle 22α (SM22α)], Extra Domain A (EDA) of Fibronectin, N-cadherin, Vimentin, Fibroblast Specific Protein 1 (FSP1), Fibroblast Activating Protein (FAP), and Calponin are upregulated (27, 213) (see Figure 4).

Inflammatory mediators such as IL1β, TNFα, and NFκB cause EC dysfunction, leading to endoMT, which is the link between inflammation and EC inflammation-associated disorders (212). Recent studies have shown that ECs can also undergo a transient stage called partial or intermediate endoMT (see Figure 4) before being completely transformed into mesenchymal cells (214, 215). Tombor and colleagues showed that ECs are transiently transformed into mesenchymal-like cells with mesenchymal gene expression three days after myocardial infarction but return to their baseline phenotype within 14 days (214). “Transitioning” cells detected in human plaques co-expressed EC and mesenchymal genes involved in endoMT (216).

#### 5.1.1. EndoMT contributes to atherosclerosis

EndoMT is essential during embryonic cardiac development and wound healing. However, dysregulated endoMT has been shown to contribute to atherogenesis (208, 216–219), which can cause flow-limiting lesions and ischemia in various organs, such as the heart, brain, and limbs, leading to angina, transient ischemic attacks and intermittent claudication. Plaque rupture, which can lead to acute ischemic syndromes such as myocardial infarction and cerebrovascular attack, is a common mechanism underlying atherosclerosis and driven by thinning of the fibrous cap induced by MMPs (220, 221). EndoMT-derived fibroblast-like cells are commonly present in atherosclerotic plaques and have been associated with plaque stability and rupture, as shown by Evrard and colleagues using EC-specific lineage tracing (212, 221–224). *In vitro* studies have revealed that various processes contributing to atherosclerosis, including TGFβ signaling, oxidative stress, and hypoxia, can activate endoMT (216).

#### 5.1.2. EndoMT contributes to the development of CAFs

Myofibroblasts and perivascular mesenchymal cells, such as pericytes, have been implicated in cancer development and progression by promoting growth, metastasis, and chemotherapy resistance (210). CAFs in the tumor microenvironment (TME) secrete a variety of growth factors (EGF, HGF, IGF1, SDF1), cytokines (IL1, IL6, IL8, IL11, LIF), chemokines, and proangiogenic factors (VEGFA, SDF1, FGF2, IL8, PDGFC), which can cause a rewiring of cellular metabolism, provoke a SASP status in senescent cancer cells, and ultimately promote cancer progression by transforming the TME into a dense and fibrotic structure (210).

Zeisberg and colleagues have shown that TGFβ1 can promote endoMT in proliferating ECs, leading to upregulation of the mesenchymal marker FSP1 and downregulation of CD31/PECAM1. Furthermore, up to 40% of CAFs in murine models of melanoma and pancreatic carcinoma are derived from ECs via endoMT (225, 226). Anti-angiogenic therapy has also been shown to have a direct effect on reducing myofibroblasts and potentially hindering cancer progression (213, 225).

In atherosclerotic CVD, endoMT promotes the accumulation of mesenchymal cells in the sub-EC spaces, which contribute to the development of fibrous plaques. Similarly, in cancer, endoMT can promote tumor invasion and metastasis by enhancing cancer cell migration and invasion and increasing the number of cancer stem cells. Furthermore, endoMT has been implicated in cancer therapy resistance. Therefore, understanding the molecular mechanisms that regulate endoMT in atherosclerotic CVD and cancer survivors could lead to the development of novel therapies to prevent both diseases (220, 221).

### 5.2. Mechanisms of endoMT-induced atherosclerosis and cancer

There are various shared pathways in cancer and atherosclerosis development. These include TGFß signaling (227, 228), bone morphogenetic proteins (BMPs) (229), and NOTCH (230). SUMOylation appears to regulate the TGFß (231) and SMAD (232) pathways and contribute to endoMT as well as both cancer and atherosclerotic CVD development and progression.

EndoMT-induced atherosclerotic CVD and cancer share several mechanisms that contribute to disease progression. Both diseases involve the accumulation of fibroblast-like cells that contribute to disease development and progression (233–239). In atherosclerosis, endoMT-derived fibroblast-like cells contribute to plaque instability and rupture (220, 221, 233–238), while in cancer, CAFs promote tumor growth, angiogenesis, and chemotherapy resistance (240, 241). Both diseases are associated with chronic inflammation, which can promote endoMT in atherosclerosis and CAF activation in cancer (242–245). Both diseases involve alterations in cellular metabolism, with endoMT and CAF contribute to the rewiring of cellular metabolism to promote disease progression (246–248). Finally, both diseases involve dysregulation of signaling pathways such as TGFβ, BMPs, NOTCH, which promotes endoMT in atherosclerosis and is a key regulator of CAF activation in cancer (211, 227, 228).

#### 5.2.1. TGFβ

**Figure 5:**
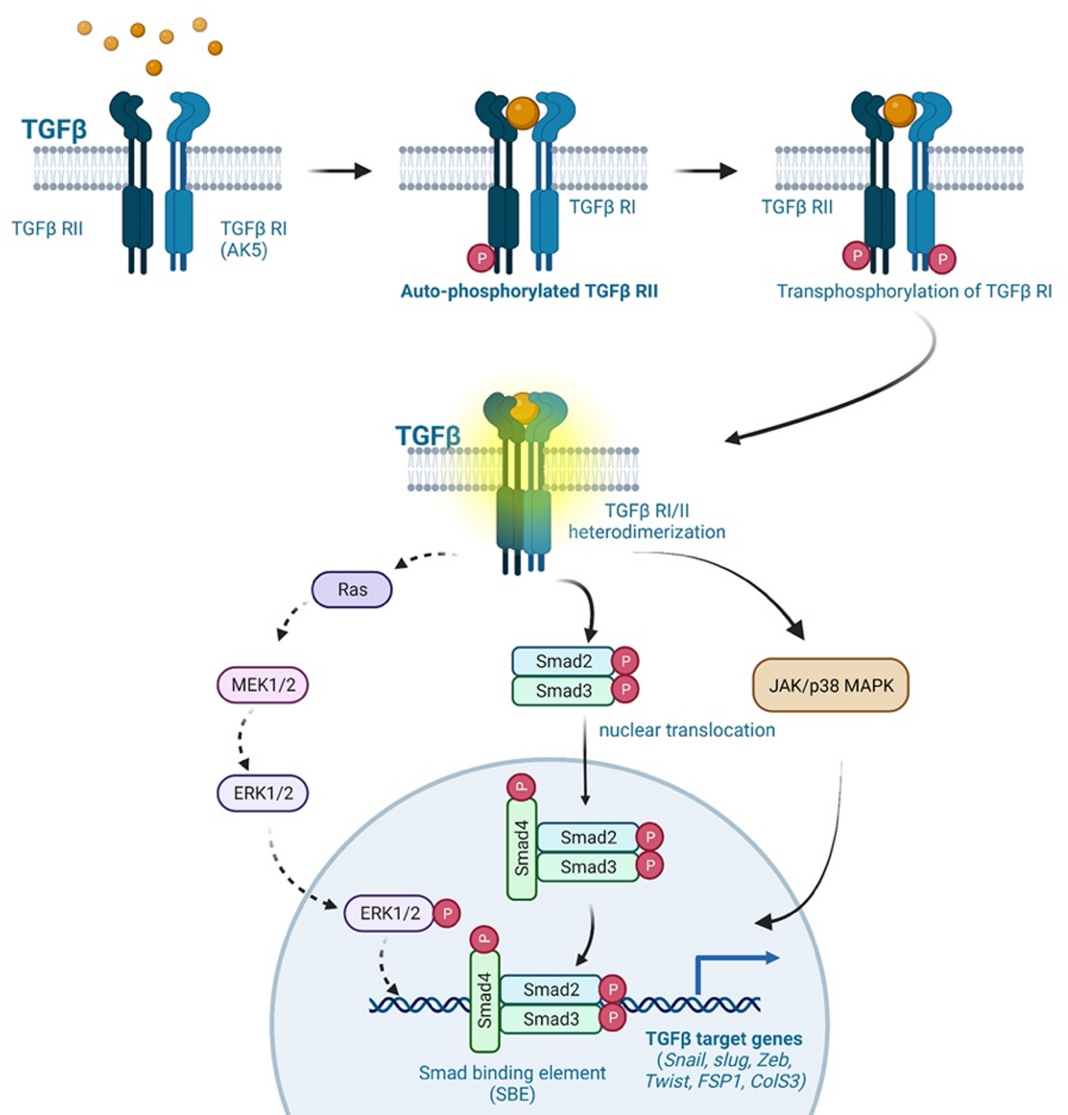
TGFβ signaling in endoMT. The TGFβ signaling pathway plays a crucial role in endoMT. Cancer cells secrete TGFβ ligands, which bind to TGFβR1 (ALK5) and TGFβR2 receptors present on ECs. This ligand-receptor binding initiates the formation of TGFβR1-TGFβR2 heterodimers, leading to the autophosphorylation of TGFβR2. Phosphorylated TGFβR2, in turn, phosphorylates TGFβR1, resulting in the activation of the TGFβR1/2 complex. The activated TGFβR1/2 complex transmits signals through both SMAD-dependent and SMAD-independent pathways. In the SMAD-dependent pathway, cytoplasmic SMAD2/3 proteins are phosphorylated by the activated TGFβR1/2 complex. Phosphorylated SMAD2/3 proteins then translocate to the nucleus, where they bind to specific SMAD binding elements (SNAIL, SLUG, ZEB, TWIST, FSP1, COLS3). This binding regulates the expression of genes involved in endoMT. In the SMAD-independent pathway, the activated TGFβR1/2 complex can also propagate signals through the activation of ERK, p38MAPK, and JNK signaling pathways. These pathways contribute to TGFβ-mediated effects on endoMT, CVD, fibrosis, and cancer progression. Overall, TGFβ signaling activation induces endoMT and is involved in various pathological processes, including CVD, fibrosis, and cancer progression (All the figures were made in Biorender.com).

Numerous types of cancer, including melanoma, esophageal carcinoma, and colon carcinoma secrete the soluble factor TGFβ (211, 227, 228). Inflammatory cytokines and d-flow inhibit FGFR1 expression in ECs, leading to activation of the TGFβ pathway, as reported by Chen and colleagues (208). Activated TGFβ signaling upregulates the expression of transcription factors (SNAIL, SLUG, and ZEB1), which, in turn, upregulates the expression of mesenchymal genes (αSMA orSM22α, calponin, vimentin, type I collagen, fibronectin, FSP1, N-cadherin, and MMP2/9) (212). TGFβ ligands secreted by TGFβ-producing cells bind the transmembrane TGFβRs, which are comprised of TGFβR1 (or ALK5) and TGFβR2 (213). This ligand-receptor association leads to the formation of the TGFβR1/2 heterodimer and the subsequent TGFβR2 auto-phosphorylation, which trans-phosphorylates TGFβR1 at specific sites, forming the active TGFβR1/2 complex. Activated TGFβR1/2 phosphorylates cytoplasmic small mothers against decapentaplegic SMAD2/3, promoting the nuclear translocation of SMAD2/3, where it binds the SMAD binding element of TGFβ target genes (such as SNAIL, SLUG, ZEB, TWIST) to upregulate their transcription (see Figure 5) (213, 249). In a SMAD-independent pathway, activated TGFβ signaling leads to the activation of the mitogen-activated protein kinase (MAPK) family of serine/threonine-specific protein kinases (ERK1/2, p38MAPK, JNK, and ERK5) (213). Medici and colleagues reported that TGFβ inhibition leads to the activation of SMAD, MEK (MAPK/ERK kinase), phosphoinositide 3-kinase (PI3K) and p38MAPK pathways, suppressing TGFβ-mediated endoMT via downregulating SNAIL expression (250). These findings suggest that activated TGFβ signaling plays a critical role in atherosclerotic CVD, cancer, and other fibrotic diseases by activating endoMT and fibroproliferative pathways (213).

#### 5.2.2. BMPs

More than a dozen members of the BMP family, which belong to the TGFβ superfamily, have been identified. The balance between BMP and TGFβ is critical for maintaining tissue homeostasis. BMP expression is altered in various types of cancer, including hepatocellular, renal, colorectal, lung, breast, prostate, endometrial, and head and neck cancers (251). BMP binds BMP cell surface receptor type 1/2 (BMPR1/2) and activates serine/threonine kinases, playing multifunctional roles in different cell types (251, 252). Following ligand-receptor binding, BMPR2 oligomerizes with BMPR1(ALK1) and phosphorylates SMAD1/5/8. In ECs, BMP binds BMPR2 and suppresses TGFβ-induced endoMT. In patients with pulmonary arterial hypertension, loss of EC BMPR2 leads to the formation of a mixed heterodimeric BMPR1/TGFβR1/TGFβR2 complex, which activates downstream TGFβ-SMAD1/5 signaling. Activated TGFβ induces SMAD2/3 signaling by a high-affinity binding to the TGFβR2-TGFβR1(ALK5) heterodimer. Additionally, TGFβ activates a lateral SMAD1/5 signaling by complexing with TGFβR2/TGFβR1/BMPR1 (ALK1) and inducing endoMT (229, 253, 254).

#### 5.2.3. NOTCH

NOTCH signaling plays important roles in both the development and pathology of the cardiac system. Mammals possess four receptors (NOTCH1/2/3/4) and five ligands (delta-like ligand 1/3/4 (DLL1/3/4) and Jagged 1/2 (JAG1/2)) (255). Upon ligand-receptor binding, enzymatic cleavages by ADAM10 and γ-secretase generate the NOTCH intracellular domain, which translocates to the nucleus, binds to the transcription factor CSL, and activates the transcription of NOTCH target genes (256). Recent studies have suggested the involvement of NOTCH signaling in endoMT (230). Noseda and colleagues have reported that EC NOTCH activation downregulates the expression of EC-specific genes (VE-cad, Tie1, Tie2, PECAM1, eNOS) and upregulates the expression of mesenchymal-specific genes (αSMA, fibronectin, PDGFRs), suggesting its role in endoMT development. This finding is supported by another study by Niessen and colleagues, demonstrating that EC NOTCH activation induces SLUG expression and endoMT (257). Additionally, NOTCH signaling has been implicated in atherosclerosis (258). TGFβ and NOTCH signaling act in a synergistic fashion during endoMT development, with TGFβ upregulating expression of NOTCH target genes such as JAG1, the receptor NOTCH1, N1ICD, and recombination signal binding protein J kappa (RBPJK)) (27, 259).

### 5.3. SUMOylation regulates endoMT-associated signaling

SUMOylation is involved in regulating many essential cellular processes, including those in the nucleus such as transcriptional activity, chromatin remodeling, and DDR (231, 260–263). It also occurs in the cytoplasmic compartment, particularly at intracellular membranes (264). One example of this is the regulation of the dynamin related GTPase DRP1, which mediates mitochondrial fission upon recruitment to the outer mitochondrial membrane (265, 266). Dysregulation of DRP1 SUMOylation affects mitochondrial division and has been associated with brain ischemia (267). Another important substrate of SUMOylation is the cystic fibrosis transmembrane conductance regulator (CFTR) is. Normally, CFTR resides in the plasma membrane, but its most common mutant form associated with cystic fibrosis contains a destabilizing phenylalanine deletion at position 508 (ΔF508) that causes protein degradation at the endoplasmic reticulum membrane (268). The degradation of ΔF508 is mediated by the ubiquitin-proteasome pathway but also involves SUMOylation (269). SUMOylation modulates the activity of multiple ion channels, including Kv7 potassium channels in hippocampal neurons linked to epilepsy and sudden death (270). Additionally, studies have shown SUMOylation regulates endoMT-associated signaling pathways, which are involved in the pathological progression of several diseases, including atherosclerosis and cancer.

#### 5.3.1. TGFβ

TGFβ signaling is regulated at various levels, including TGFβR activation and post-translation modifications (271). TGFβ SUMOylation plays a critical role in epithelial to mesenchymal transition, which is similar to endoMT (231). As studies have shown that flow exerts profound effects on SUMOylation in atherosclerotic CVD (262, 272), we discuss the potential contributions of TGFβ SUMOylation to endoMT under flow conditions.

The TGFβ signaling cascade begins with TGFβR1/2 dimerization followed by TGFβ activation. Activated TGFβR1/2 promotes TGFβR1 K389 SUMOylation, which enhances TGFβR-SMAD association and SMAD2/3 phosphorylation (273). TGFβR SUMOylation also increases the metastasis and invasiveness of tumor cells. SENP2 inhibits TGFβR1 SUMOylation, thus suppressing TGFβ-induced epithelial to mesenchymal transition in bladder cancer (274). SENP2 overexpression in invasive bladder cancer cells, T24, suppresses TGFβ-mediated SMAD2/3 phosphorylation, downregulates the expression of mesenchymal markers N-cadherin and fibronectin, and inhibits epithelial to mesenchymal transition (274). Although we have reported the role of d-flow-induced SENP2 nuclear export on SUMOylation of ERK5 and p53 (263), there are no reports on flow effects on SENP2-regulated TGFβ/SMAD signaling. This area can be a subject of future studies.

#### 5.3.2. SMAD

**Figure 6:**
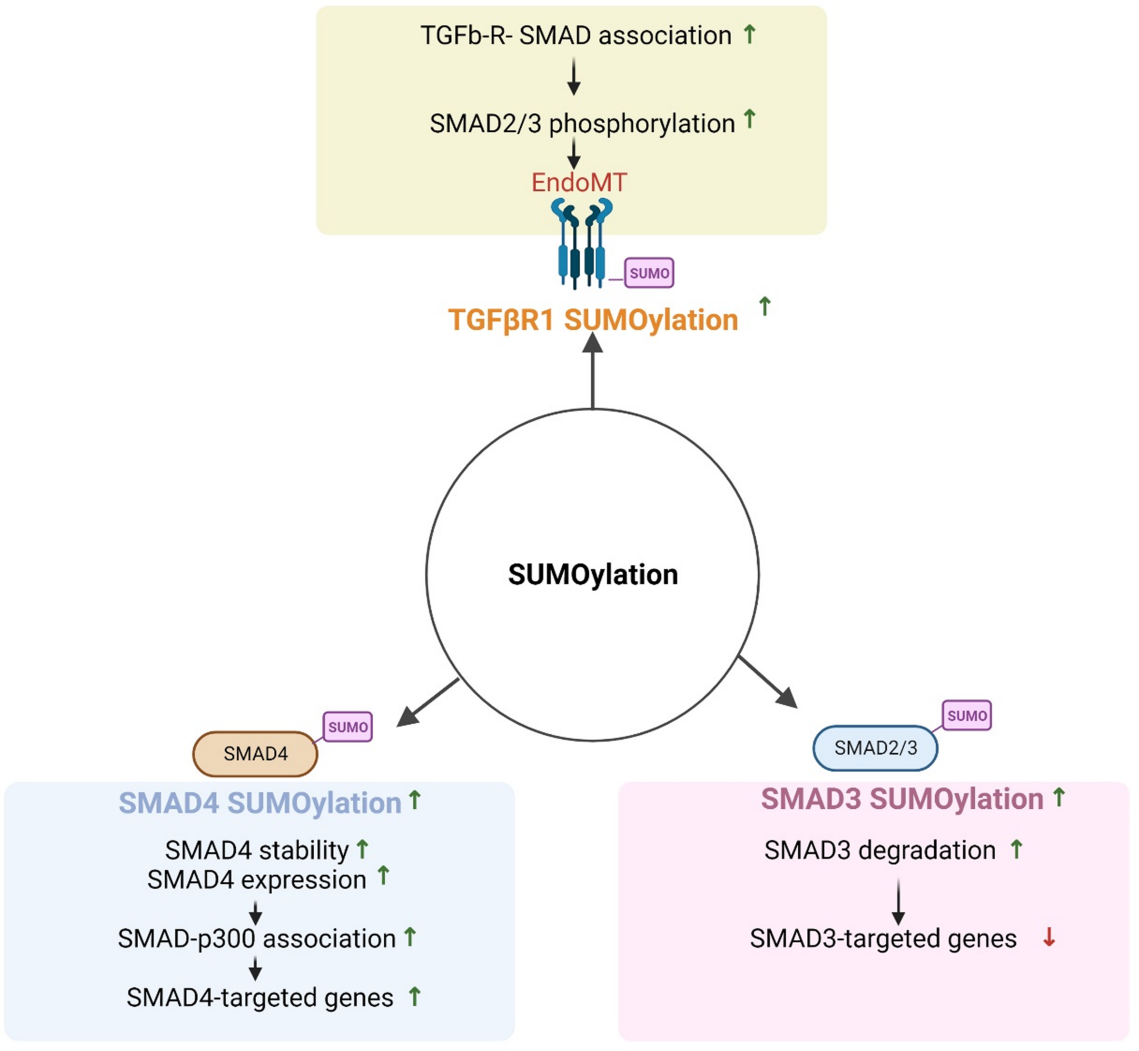
Regulation of TGFβ signaling by SUMOylation (All the figures were made in Biorender.com).

The SMAD family of proteins is divided into three subfamilies: receptor-activated SMADs (r-SMADs, including SMAD1/2/3/5/8), common mediator SMADs (Co-SMADs), and inhibitory SMADs (iSMADs including SMAD6/7) (275). R-SMADs are activated by specific ligand-receptor binding, such as TGFβR, and play a crucial role in transmitting signals from the receptor to the nucleus. iSMADs act as negative regulators of TGFβ signaling by inhibiting r-SMAD activation (276). rSMAD SUMOylation can modulate their function and activity by altering their subcellular localization, stability, and interaction with other proteins. For example, SUMOylation of SMAD4 at K113 and K159 increases its transcriptional activity by promoting interaction with histone acetyltransferase p300. SENP1/2 regulate TGFβ-induced epithelial to mesenchymal transition by inhibiting SMAD4 SUMOylation (277, 278). SMAD3 SUMOylation inhibits its transcriptional activity by promoting degradation. iSMAD SUMOylation can also modulate their function and stability; for instance, SMAD7 SUMOylation increases its inhibitory activity by promoting its stability and preventing proteasome-mediated degradation, while SMAD6 SUMOylation promotes its degradation and reduce its inhibitory activity (see Figure 6).

## 6. The role of blood flow in cellular senescence and the development of atherosclerosis in cancer survivors

ECs play a crucial role as transducers between blood flow mechanics and cellular signaling pathways in both physiological and pathological conditions. Mechano-sensors on the surface of ECs sense changes in blood flow and transduce these mechanical forces into biochemical signals, subsequently activating cellular responses that regulate downstream events (279). Hemodynamic shear stress, which is the frictional force generated by intraluminal blood flow on the surface of ECs, influences the formation of atherosclerotic plaque. Hemodynamic stress is expressed as a force per wall area (280, 281), but different patterns of shear stress can occur depending on the morphology of blood vessels. These patterns can be categorized as either linear or laminar flow (l-flow, also referred to as s-flow or u-flow in our previous publications) (282, 283) or d-flow, which is characterized by the recirculation of blood in vessel bends, branches, and bifurcations. These distinct flow patterns can have different effects on the secretory function and morphology of ECs. L-flow and d-flow have opposite effects on ECs and have been implicated in various cellular processes (71, 213, 284–286).

### 6.1. Blood flow in atherosclerosis

#### 6.1.1. L-flow inhibits atherosclerosis

In regions of l-flow, ECs aligned longitudinally in the direction of blood flow and secrete factors for vasodilation and anticoagulation. L-flow promotes the generation of NO and expression of downstream molecules that improve vascular injury and suppress inflammation, making it an atheroprotective mechanical force that maintain EC homeostasis and function (191). This is accomplished in part through the activation of extracellular-signal regulated kinase 5 (ERK5), which increases endothelial nitric oxide synthase (eNOS) expression and inhibits EC inflammation (272, 287).

Studies on cultured human aortic ECs have shown that l-flow downregulates proliferation- and inflammation-related genes and upregulates survival-, angiogenesis- (e.g., tyrosine-protein kinase receptor Tie2 and vascular endothelial growth factor receptor Flk1) and vascular remodeling- (e.g., MMP1) related genes (288). This suggests that l-flow maintains a non-proliferative and non-inflammatory EC gene expression profile.

L-flow increases NO levels immediately through stimulation of eNOS, and long-term exposure can lead to increased mRNA and protein expression of eNOS. Acute eNOS activation could result from a shear-stress-derived intracellular calcium increase, which enhances calmodulin binding to eNOS. Nonetheless, eNOS phosphorylation by various phosphorylases, including PI3K/AKT, adenylate cyclase, and protein kinase A (PKA) have also been involved in the rapid response to l-flow (289). Long-term l-flow-dependent eNOS activation has been linked to SIRT1, a histone deacetylase, and its interaction with AMPK, as well as to various transcription factors that ultimately upregulate eNOS expression, such as KLF2 (290).

Furthermore, the resulting high shear stress has been found to increase BH4 levels by stimulating the first and rate-limiting step of its synthesis, GCH1. L-flow resulted in a ∼30-fold increase in GCH1 activity produced through phosphorylation of serine 81 by the α' subunit of casein kinase 2 (CK2), but did not produce increased protein expression, indicating BH4's short-term role in l-flow-associated NO synthesis. This, in turn, increases levels of NO and promotes an overall cardioprotective effect (291).

#### 6.1.2. D-flow promotes atherosclerosis

**Figure 7:**
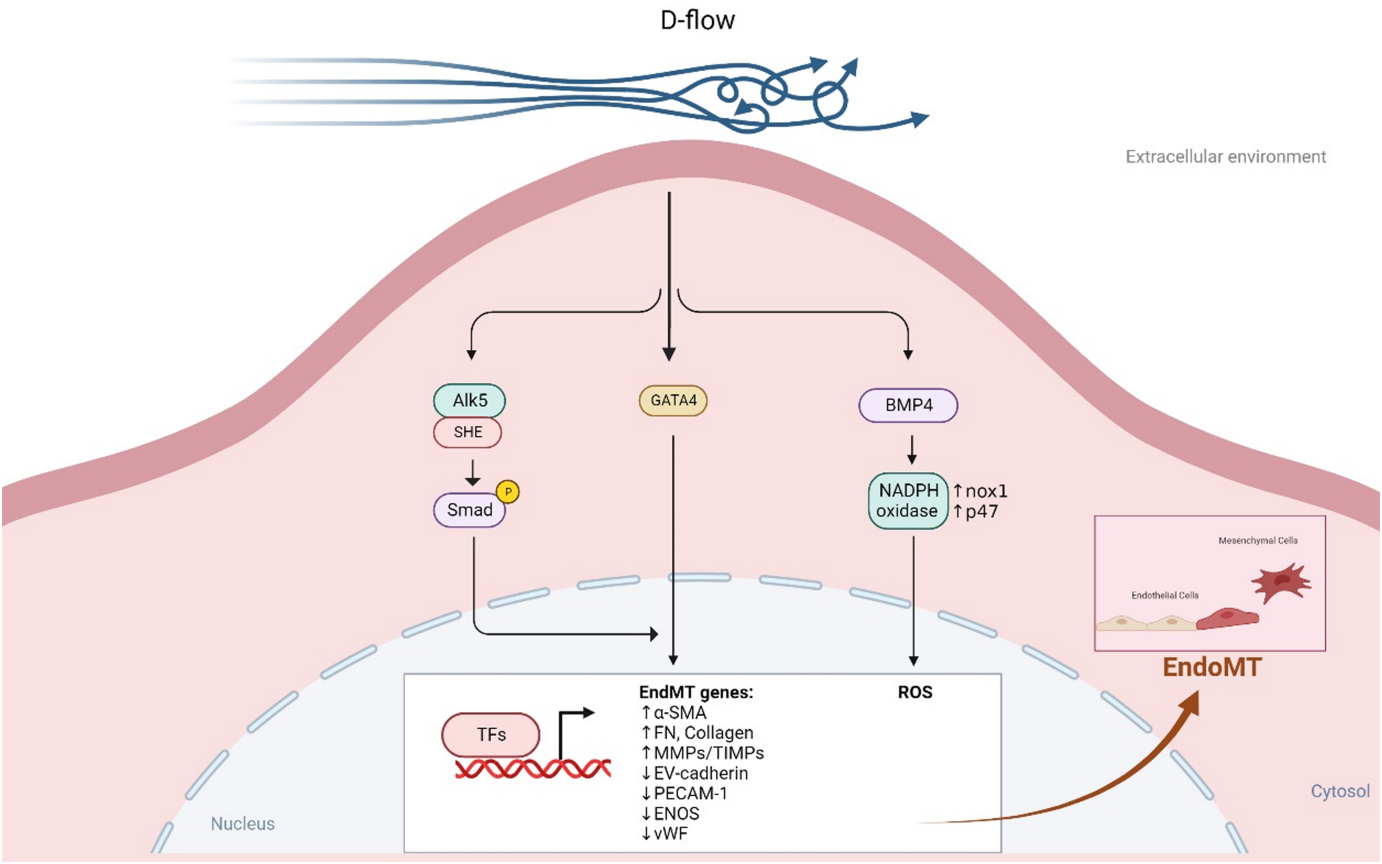
D-flow promotes endoMT (All the figures were made in Biorender.com).

In regions of d-flow, ECs undergo several changes including becoming polygonal and poorly aligned and undergo senescence and apoptosis. Moreover, they also secrete factors that lead to vasoconstriction and coagulation, promoting the generation of ROS, platelet aggregation, EC activation, senescence, inflammation, dysfunction, permeability, and endoMT as depicted in Figure 7 (207, 292, 293). This disruption of EC homeostasis (294) and proatherogenic signaling can worsen atherosclerotic CVD (207, 292). D-flow is a well-known stress stimulus that can cause vascular senescence, and several mechanisms such as endoMT (208), NO synthesis, telomere shortening (295, 296), oxidative stress (188), and chronic inflammation (297) have been proposed to explain how d-flow mediate atherosclerotic plaque formation (293, 298–302).

#### 6.1.3. D-flow induces endoMT

D-flow and l-flow have different effects on the accessibility of transcription factor binding motifs and cis-regulatory elements, leading to differential regulation of EC-specific gene expression at both genomic and epigenomic levels. Downstream factors, such as kruppel-like factors 2/4 (KLF2/4), are enriched in ECs exposed to l-flow, while known transcription factor binding motifs (RELA, Fos/Jun, and TEAD1) and novel transcription factor binding motifs (TEF, ETV3, and STAT1) are enriched in ECs exposed to d-flow (286).

Research using single-cell RNA-seq and single-cell assay for transposase accessible chromatin sequencing (ATACseq) in mice after partial carotid ligation showed that d-flow can reprogram ECs from athero-protective cells to atherogenic proinflammatory cells, endoMT cells, hematopoietic stem cells, endothelial progenitor cells, and cells with immune cell-like phenotypes (286). Mechanistically, d-flow can activate the TGFβR1 (ALK5) mechano-sensor in ECs, leading to downstream activation of molecules involved in d-flow-mediated endoMT, including TGFβR1-associated Src homology and collagen (Shc) (279). D-flow also promotes BMP4 production through Nox1-based NADPH oxidase, subsequently increasing ROS synthesis, a potential activator of endoMT (213, 303, 304) (see Figure 7). By contrast, l-flow prevents endoMT by upregulating the expression of extracellular matrix protein tenascin-X through KLF4 in mouse and human ECs, which binds TGFβ and hinders TGFβ-TGFβRs binding, thereby suppressing TGFβ-induced endoMT and atherogenesis (305).

Through a series of *in vivo* and *in vitro* studies, Mahmoud and colleagues have demonstrated that d-flow upregulates the expression of twist family bHLH transcriptional factor 1 (TWIST1) via a mechanism regulated by the developmental transcription factor GATA4. Microarray data revealed that both GATA4 and TWIST1 expression were enriched in ECs exposed to d-flow regions in the porcine aorta *in vivo*. *In vitro* studies using controlled flow systems also showed that GATA4 and TWIST1 expression were enhanced in cultured ECs exposed to d-flow compared to non-d-flow conditions (306, 307). Activation of the GATA4-TWIST1 pathway by d-flow upregulated SNAIL, a downstream transcription factor of TWIST1, and a positive regulator of endoMT markers such as SLUG, N-cadherin, and αSMA. These events subsequently induce proliferation, inflammation, permeability, and endoMT in ECs exposed to d-flow, leading to atherogenesis (308).

Chen and colleagues have found that d-flow, along with soluble inflammatory factors (IFNγ, TNFα, and IL1β), can decrease the expression of fibroblast growth factor receptor 1 (FGFR1) in ECs, leading to activation of downstream TGFβ signaling and subsequent endoMT activation (208). Studies in patients with coronary artery diseases have shown that disease severity correlates with FGFR1 expression, TGFβ signaling activation, and the extent of endoMT activation. EndoMT activation enhances atherosclerotic plaque formation by modulating fibronectin deposition and upregulating VCAM1 and ICAM1, which in turn recruit circulatory monocytes and leukocytes while generating new mesenchymal cells (208, 309). The extent of endoMT activation is positively correlated with the unstable phenotype of plaques, which can be driven by increased synthesis of collagen-MMPs by endoMT-derived fibroblast-like cells in atherosclerotic plaques (216).

Zhao and colleagues used a three-dimensional micro-engineered human coronary artery-on-a-chip model to demonstrate that d-flow drives ECs towards a proinflammatory endoMT phenotype, accompanied by elevated expression of VCAM1 and ICAM1, which can mediate recruitment of monocytes, leukocytes, and macrophages to plaque sites (310, 311). Activated macrophages and T lymphocytes release cytokines, which further increase MMP production (312), leading to degradation of the extracellular matrix and ultimately causing plaque ruptures. Macrophages can also directly induce apoptosis of vascular smooth muscle cells (313–315), which are responsible for collagen I and III production, critical factors for plaque stability and repair (312, 316). All these processes work together to prime atherosclerotic plaques for rupture (312, 317).

#### 6.1.4. D-flow alters NO signaling

Altered synthesis or activity of NO and oxidative stress play a crucial role in d-flow-induced EC dysfunction (318). The levels of BH4 is reduced by d-flow (291) and aging (181, 188), leading to the production of superoxide (O_2_^–^), a potent ROS, and pro-atherosclerotic vascular dysfunction (191). In the absence of BH4, NO synthesis can result in the production of superoxide from the ferrous-dioxygen complex, leading to eNOS uncoupling, which could explain why d-flow produces ROS but decreases NOS (191).

### 6.2. Blood flow in cancer metastasis

A high-resolution model was developed to simulate the spread of circulating tumor cells (CTCs) through the bloodstream. The model includes stochastic adhesion events and uses a realistic model of global blood circulation to simulate cancer cell trajectories. The authors compared the model's predictions with data from thousands of human autopsies for seven different solid tumors, including lung, prostate, pancreatic, and colorectal cancers. They found that, on average, 40% of the variation in the metastatic distribution could be attributed to blood circulation (319–328).

### 6.3. D-flow promotes endoMT through SUMOylation

Activation of the transcription factor NFκB is critical for initiating an inflammatory genetic program critical that contributes to atherogenesis, which is triggered by d-flow (263, 272, 283, 329, 330). Ganguli and colleagues investigated the mechanisms underlying NFκB activation and found that d-flow regulates NFκB by modulating Ras-GTPase (331). Furthermore, Mabb and colleagues demonstrated that in response to genotoxic stress stimuli, the signal transducer and activator of transcription (STAT) activates NFkB through SUMOylating the NFkB essential modulator (NEMO) (332).

D-flow induces various posttranslational modifications that promote inflammatory signaling, including SUMOylation (262). SUMOylation is a reversible and dynamic process that occurs in approximately 20% of proteins and is associated with various diseases such as atherosclerosis and cancer (262, 263, 333–336). This process requires the activity of sentrin-specific proteases (SENPs) that catalyze the covalent attachment and detachment of SUMO to and from Lysine (K) residues on substrates (337). Six SENP isoforms have been identified (SENP1/2/3/5/6/7), each with substrate specificities and subcellular localizations. Among these isoforms, SENP2 binds NEMO, inhibits NEMO SUMOylation (338), and suppresses DNA damage-induced NFkB activation (339).

Knockout of SENP2 in mice leads to developmental defects in trophoblast stem cell niches and lineages due to dysregulation of the Mdm2-p53 signaling pathway in the placenta (340). In human hepatocellular carcinoma, SENP2 acts as a tumor suppressor by modulating β-catenin stability (341). Similarity, in osteosarcoma cells, SENP2 negatively regulates proliferation, migration, and invasion (342). It is important to note that NEMO is not the only substrate of SENP2, given its broad deSUMOylation activity (343). SENP2 contains multiple nuclear localization and export signals, allowing it to shuttle between the nucleus and cytoplasm (344), a process regulated by posttranslational modifications (263, 334). Depletion of SENP2 increases SUMOylation of ERK5 and p53 (338), leading to EC inflammation and apoptosis, respectively (263).

## 7. Possible molecular mechanisms for the development of atherosclerosis in cancer survivors

### 7.1. Both d-flow and cancer treatment can accelerate premature senescence

Atherogenesis predominantly occurs in regions of vascular ECs exposed to d-flow but not l-flow (287, 336, 337, 345–349). ECs exposed to d-flow exhibit accelerated generation of ROS, telomere shortening, and telomere dysfunction, leading to SIPS (71, 295–297, 350–352). During atherogenesis, senescent cells accumulate in d-flow regions as shown by Warboys and colleagues. They demonstrated that d-flow induces premature senescence via activation of the p53-p21 signaling pathway, which is attenuated by pharmacological activation of sirtuin 1 (SIRT1) (352).

Similarly, many cancer treatments, including radiation therapy and chemotherapy, can induce cellular senescence, a phenomenon known as TIS (206, 353, 354). While non-senescent inflammatory cells secret cytokines that activate a temporary inflammatory stage in ECs, subsets of cancer treatment-induced senescent cells secrete proinflammatory cytokines, chemokines, growth factors pro-angiogenic factors, and ROS, known as SASP, that promote inflammation and aberrant cell growth (10–12, 68, 355–357). SASP can be induced by both cancer treatment and d-flow and can produce long lasting inflammation (PISP) that instigates atherogenesis. Therefore, SASP may be an important target for mitigation strategies (79, 354, 358).

### 7.2. Both d-flow and cancer treatment can reduce BH4

Similar to d-flow (291), cancer therapy (188) reduce the levels of BH4 (181). Radiation creates an oxidative environment, which promotes the oxidation of BH4 into BH2 and other oxidized byproducts, causing the uncoupling of eNOS and leading to an increase in superoxide synthesis. BH4 deficiency, eNOS uncoupling, and vascular dysfunction can occur in irradiated cells due to the downregulation of GCH1, the enzyme that catalyzes the second step in BH4 *de novo* biosynthesis (359). The decreased BH4:BH2 ratio resulting from d-flow produces pro-atherosclerotic vascular dysfunction (181, 360, 361). EC dysfunction resulting from changes in the BH4:BH2 ratio and subsequent eNOS uncoupling is a characteristic feature of various diseases, including atherosclerosis.

### 7.3. Both d-flow and cancer treatment can activate p90RSK

Different flow patterns activate distinct kinase pathways that affect EC morphology and function. L-flow transiently activates both AMP-activated protein kinase (AMPK) and protein kinase B (PKB, or AKT), while d-flow activates only PKB (362, 363). In addition, our studies have shown that p90RSK, a redox sensitive kinase, is specifically activated not only d-flow, but not l-flow. Moreover, we have also demonstrated that p90RSK is activated not only by d-flow but also by chemotherapy and IR, suggesting its potential role on mediating cellular response to these stimuli (88, 287, 335, 364).

Radiation therapy is a common treatment modality for solid tumors (365), with over 30% of cancer patients in the United States receiving radiation therapy as part of their treatment plan, often combined with chemotherapy, immunotherapy, or surgery as first-line therapy (366). However, thoracic radiotherapy can result in delayed CVD development (367). Studies have shown that radiation can cause endoMT, which contributes to the development of atherosclerotic CVD and CAFs in cancer (209, 368–370), as discussed above.

To investigate the molecular mechanism underlying atherosclerotic CVD development after thoracic radiotherapy, Kim and colleagues exposed human aortic ECs to 5Gy of IR for 24 h and observed an increased expression of fibroblast markers αSMA and FSP1 accompanied by a decreased expression of EC markers CD31 and VE-cad. The authors also noted that oxLDL increases IR-induced endoMT, which contributes to atherosclerotic plaque formation (209). Similarly, irradiation of human pulmonary artery ECs induces endoMT with an increased expression of hypoxia inducible factor 1α (HIF1α) and activation of TGFβR1/SMAD signaling (368).

Recent research by Choi and colleagues reported that in human umbilical vein ECs exposed to radiation, TRP53 expression increases while TGFβ2 decreases endoMT. siRNA-mediated TRP53 silencing abrogated radiation-induced endoMT, resulting in a decreased expression of endoMT-related transcription factors such as SNAIL1/2 and zinc finger E-box-binding homeobox 2 (ZEB2) (370). These findings highlight the complex interplay between radiation-induced signaling pathways and the development of atherosclerotic CVD in cancer survivors. Furthermore, as 30hosphory earlier, p90RSK activation plays a potential role in mediating cellular response to radiation and chemotherapy, further underscoring its potential involvement in the development of atherosclerotic CVD in cancer survivors.

### 7.4. p90RSK activation regulates SUMOylation by driving SENP2 phosphorylation

We have found that d-flow activates p90RSK (287, 335), which phosphorylates SENP2 T368 (335). This leads to the export of SENP2 from the nucleus, causing a loss of its deSUMOylation activity within the nucleus. As a result, there is an increase in the SUMOylation of ERK5 and p53, ultimately leading to atherosclerosis. These findings help explain why atherosclerotic plaques mainly develop in vessel bends or bifurcations, where d-flow is generated (191, 291, 371, 372). Despite the growing understanding of the effects of flow on ECs (349), the mechanisms by which l-flow inhibits EC turnover, maintains EC homeostasis, and prevents atherogenesis remain incompletely understood.

Furthermore, we have discovered that cytoplasmic SENP2 plays a role in inhibiting the SUMOylation of the membrane-associated protein MAGI1, which is a tight and adherent junction protein with a newly identified role in EC function and atherosclerosis (334). Recently, we also reported that SENP2 regulates the SUMOylation of focal adhesion kinase (FAK) at K152 through a mechanism that is independent of T368 phosphorylation (373, 374). These findings suggest that the regulation of SUMOylation by SENP2 through post-translational modifications is more complex than previously thought and requires further investigation. For example, it is important to understand how the SUMOylation machinery is targeted to cell membranes and how it regulates post-modifications. Mass-spectrometry-based phospho-proteomics has revealed that SENP2 can be phosphorylated at S32, S333, and S344 residues (375, 376). However, the mechanisms underlying the31hosphorylateon of these residues by kinases activated during altered flow, as well as the associated regulatory mechanisms and biological consequences, remain to be investigated.

**Figure 8:**
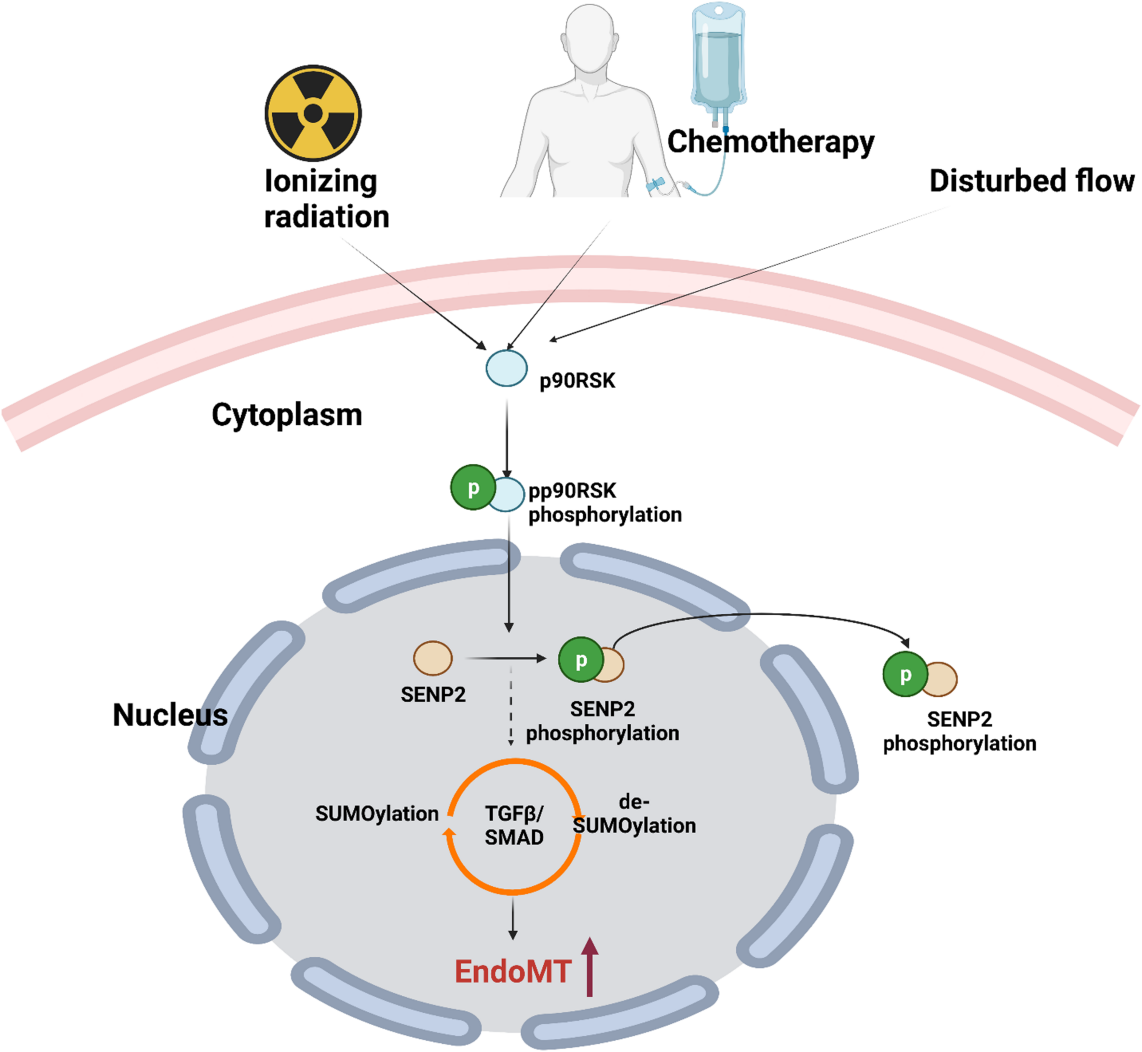
Endomt activation by d-flow, chemotherapy, or radiation therapy through the activation of p90RSK (All the figures were made in Biorender.com).

Figure 8 illustrates the plausible mechanism of endoMT development by d-flow, chemotherapy, or radiation through the activation of p90RSK.

### 7.5. p90RSK activation accelerates SIPS by driving TERF2IP posttranslational modifications

**Figure 9:**
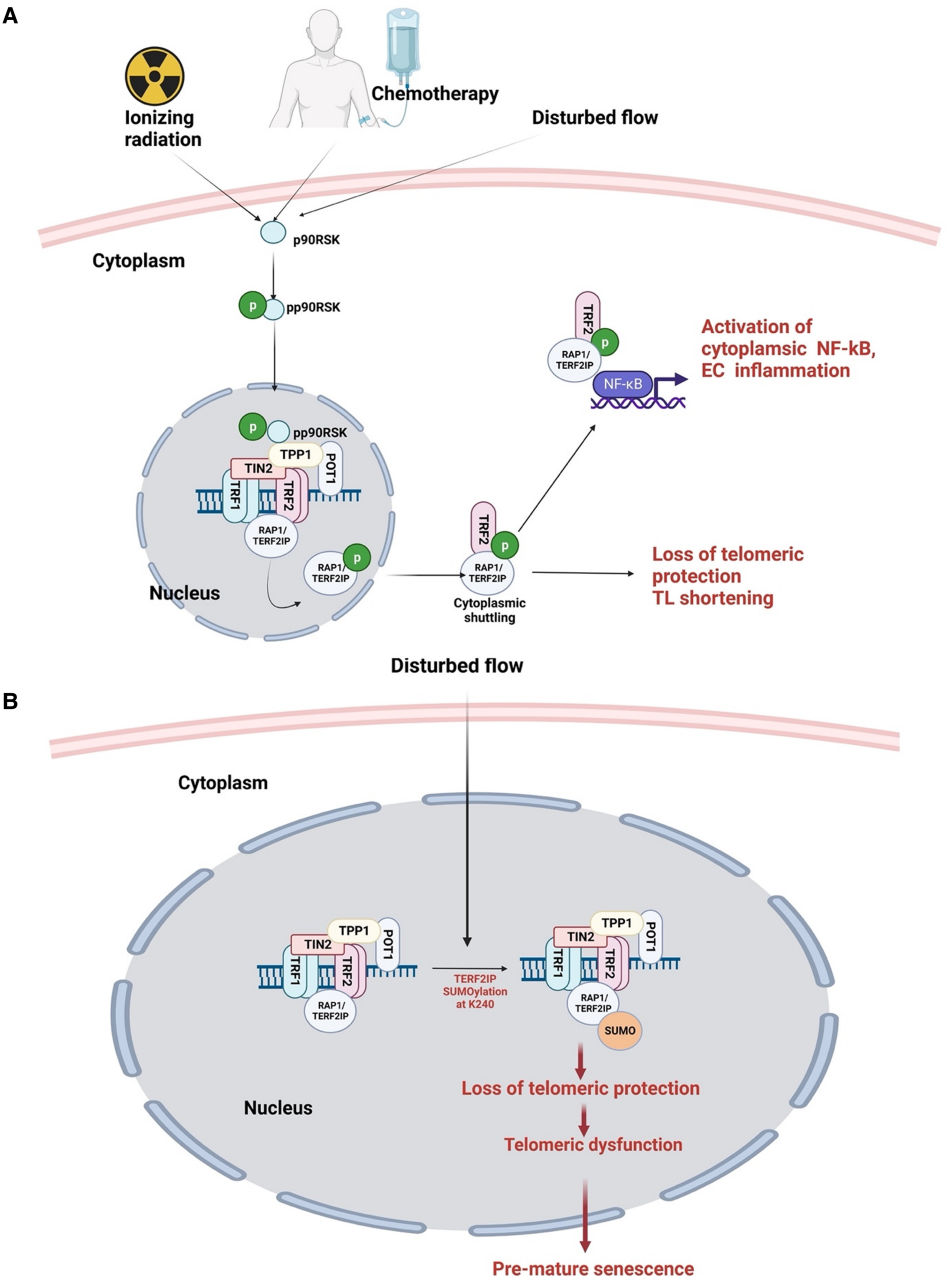
Posttranslational modifications of TERF2IP induced by d-flow and cancer treatment disrupt Shelterin and induce premature senescence. (**A**) External stimuli, such as radiation, chemotherapeutic agents, and d-flow, activate p90RSK through phosphorylation. Activated p90RSK translocates to the nucleus and phosphorylates TERF2IP (RAP1), leading to TERF2IP nuclear export and loss of telomeric protection. Unprotected telomere becomes susceptible to stress-induced damage, resulting in telomere shortening. Cytoplasmic TERF2IP activates p65 NFκB, triggering EC inflammation. (**B**) D-flow induces SUMOylation of TERF2IP at K240, causing loss of telomeric protection, telomeric dysfunction, and premature senescence (All the figures were made in Biorender.com).

Post-translational modifications are crucial for regulating the cellular localization and function of TERF2IP, which is necessary for protecting telomeres (295, 377). Our recent studies have revealed that activation of p90RSK leads to phosphorylation of TERF2IP S205. This phosphorylation event promotes TERF2IP nuclear export, which leads to the loss of TERF2IP telomeric protection and TRF2 removal from telomeric DNA, ultimately accelerating telomere shortening and cellular senescence (see Figure 9) (295). Additionally, TERF2IP can be modified by Small Ubiquitin-related Modifier (SUMO), which disrupts the TERF2IP-TRF2 association and leads to nuclear export (377). Both scenarios result in cytoplasmic TERF2IP-mediated activation of nuclear factor kappa B (NFkB) p65 subunit, ultimately leading to cellular senescence, inflammation, and atherosclerosis (295, 377–381).

Notably, TERF2IP expression is increased in d-flow regions of atherosclerotic plaques and has been linked to various cancers, such as breast cancers, gastric carcinoma, non-small cell lung cancer, and mantle cell lymphoma (106, 382–384). In metastatic colon cancer, TERF2IP activates NFkB, leading to the phosphatase of regenerating liver 3 (PRL3) activation and increased TERF2IP expression and nuclear export, ultimately promoting cancer cell invasiveness and metastasis (378–381, 385).

In a study using mice that received 2 Gy whole-body γ-radiation, it was found that VCAM1 expression is upregulated in the d-flow, further implicating the role of p90RSK-mediated TERF2IP S205 phosphorylation in the progression of atherosclerotic CVD progression induced by d-flow and chemoradiation therapies (295, 377).

## 8. Senotherapies for the treatment of age-related diseases

The accumulation of senescent cells during RS or SIPS is one of the challenges in the treatment of atherosclerotic CVD and other age-related diseases. Therefore, eliminating senescent cells using senolytic drugs or attenuating the SASP without inducing apoptosis of senescent cells using senomorphic drugs is considered a potential treatment strategy nowadays. Table 5 lists senotherapies commonly used in clinical or preclinical settings.

**Table 5:** List of senotherapies used for age related diseases (386).

Name of senotherapy	Targeted diseases	Reference
Quercetin	Coronary artery disease	Clinical trial: NCT04907253
Curcumin	Cardiovascular diseases	Clinical trial: NCT04119752, NCT01968564
UBX0101	Degenerative diseases of the joints in the body	Clinical trial: NCT03513016
UBX1325	Age related muscular degeneration	Clinical trial: NCT04537884, NCT05275205
Fisetin	Tested in progeroid mice model to prevent premature aging	(387)
Cardiac Glycosides	Lung fibrosis	(388)

## 9. Discussion

Our aim with this review is to offer a comprehensive overview of the interactions between multiple key components that contribute to SIPS and the development of atherosclerotic CVD in cancer survivors. We intend to identify potential therapeutic targets for the prevention and treatment of atherosclerotic CVD and provide new insights into the molecular mechanisms underlying atherosclerosis development in cancer survivors. We believe that the information presented in this review will be beneficial to a diverse audience of researchers and clinicians in the fields of cardiology, oncology, and molecular biology, as well as to patients and their families who may be concerned about the long-term health effects of cancer treatment. Finally, we emphasize the need for further research, particularly in ECs, to gain a better understanding of the mechanisms involved in the development of atherosclerosis in cancer survivors after receiving cancer therapy.

A potential link between BH4 and eNOS uncoupling has been proposed, but the precise mechanism underlying this process remains unclear (188). Preventing this oxidative process may limit the impact of aging on vascular function (182). Studies investigating the acute BH4 supplementation on vascular function have shown positive short-term effects, likely by preventing eNOS uncoupling and ROS formation, leading to overall cardioprotective NO synthesis. However, chronic administration of BH4 appears to have little long-term effect, possibly due to rapid oxidation into its inactivated form, BH2 (181). A recycling pathway that converts BH2 back to BH4 through DHFR exists, and reduced DHFR levels lead to altered BH4:BH2 ratios and eNOS uncoupling-derived EC dysfunction (181). Deficiencies in NO bioavailability, in which BH4 plays a critical role, are the primary cause of age-related reduction in endothelium-dependent dilation (389). This vascular impairment is a hallmark of the physiological aging process and is also involved in the pathophysiology of age-related conditions, as well as the deleterious effects of cancer treatment on the vasculature.

The impact of altered hemodynamic shear stress, either alone and in combination with cancer treatment, on DDR signaling pathways and SIPS in cancer survivors is not well understood and requires further investigation. However, it is possible that d-flow and cancer therapy accelerate premature senescence through p90RSK-driven posttranslational modifications of TERF2IP and disruption of the Shelterin complex, which is formed by TRF1, TRF2, TIN2, TPP1, POT1, and TERF2IP/RAP1 (121, 122). Alterations in any of these Shelterin proteins cause aberrant telomere protection and often lead to a pro-senescent stress-induced phenotype associated with disease states (122).
